# Photon Upconversion Hydrogels for 3D Optogenetics

**DOI:** 10.1002/adfm.202010907

**Published:** 2021-06-04

**Authors:** Rinat Meir, Tal Hirschhorn, Sungsoo Kim, Kealan J. Fallon, Emily M. Churchill, Dino Wu, Hee Won Yang, Brent R. Stockwell, Luis M. Campos

**Affiliations:** Department of Chemistry, Columbia University, New York, NY 10027, USA; Department of Biological Sciences, Columbia University, New York, NY 10027, USA; Department of Pathology and Cell Biology, Columbia University Medical Center, New York, NY 10032, USA; Department of Chemistry, Columbia University, New York, NY 10027, USA; Department of Chemistry, Columbia University, New York, NY 10027, USA; Department of Chemistry, Columbia University, New York, NY 10027, USA; Department of Pathology and Cell Biology, Columbia University Medical Center, New York, NY 10032, USA; Department of Chemistry, Columbia University, New York, NY 10027, USA, Department of Biological Sciences, Columbia University, New York, NY 10027, USA; Department of Chemistry, Columbia University, New York, NY 10027, USA

**Keywords:** biomaterials, hydrogels, optogenetics, photon upconversion, triplet–triplet annihilation

## Abstract

The ability to optically induce biological responses in 3D has been dwarfed by the physical limitations of visible light penetration to trigger photochemical processes. However, many biological systems are relatively transparent to low-energy light, which does not provide sufficient energy to induce photochemistry in 3D. To overcome this challenge, hydrogels that are capable of converting red or near-IR (NIR) light into blue light within the cell-laden 3D scaffolds are developed. The upconverted light can then excite optically active proteins in cells to trigger a photochemical response. The hydrogels operate by triplet–triplet annihilation upconversion. As proof-of-principle, it is found that the hydrogels trigger an optogenetic response by red/NIR irradiation of HeLa cells that have been engineered to express the blue-light sensitive protein Cry2olig. While it is remarkable to photoinduce the clustering of Cry2olig with blanket NIR irradiation in 3D, it is also demonstrated how the hydrogels trigger clustering within a single cell with great specificity and spatiotemporal control. In principle, these hydrogels may allow for photochemical control of cell function within 3D scaffolds, which can lead to a wealth of fundamental studies and biochemical applications.

## 1. Introduction

The ability to modulate cell function through on-demand external triggers within 3D biomaterials has the potential to significantly transform biological studies of chemically induced signaling pathways, impacting areas of disease control/prevention, and tissue engineering.^[1–3]^ Specifically, light-responsive biomaterials provide newfound opportunities for fundamental studies in photochemical processes within synthetic matrices that mimic the native microenvironment of cells.^[4,5]^ The use of light to induce photochemical processes offers numerous, advantages due to its versatility to be, readily modulated in space and time. To date, light-activated reactions that require short-wavelength irradiation in the visible region (*λ* < 500 nm), both intracellularly or in the cell microenvironment, are mainly carried out in 2D cultures, including reactions such as polymerizations from cell surfaces^[6]^ and optogenetic processes.^[7–9]^ However, there are prohibitive limitations when inducing photochemical transformations within 3D matrices. A major obstacle lies in the need to use blue-light to induce reactions—these high-energy photons have short penetration depth through most solid-state media (e.g., cell-loaded 3D biomaterials, tissue, etc.) due to light scattering and background absorption.^[10]^ To overcome such challenges, processes that involve long-wavelength two-photon excitation have been applied to various systems, albeit with costly limitations in the use of high fluence equipment and small surface area of irradiation.^[11,12]^, Alternatively, recent breakthroughs employ unconventional photophysical processes where materials that absorb low-energy photons in the IR region produce emission of high-energy photons (photon upconversion) that can be exploited for light stimulation of neurons.^[13–15]^

There are two available photon upconversion systems that have the potential to be exploited in synthetic biomaterials due to the low power excitation required and tunable excitation/emission wavelengths: 1) photon upconversion with inorganic nanoparticles;^[13,14,16]^ or 2) the multiexciton process of triplet–triplet annihilation upconversion (TTA-UC) from organic chromophores.^[17–19]^ By implementing photon upconversion processes that can operate either by blanket and/or focused irradiation using inexpensive commercial low-energy light sources, the realization of photo-chemically induced processes in 3D will enable a wide variety of studies of biological systems.^[20,21]^ For example, a key strategy for manipulating cells with light involves fusing optogenetic proteins to various cellular proteins that can be either activated or deactivated by blue light.^[8,9]^ Thus, the ability to integrate upconversion systems within biomaterials provides a route to exquisitely control photochemically triggered processes in 3D cultures, and importantly through tissue penetration (drug delivery, degradation, cell signaling, etc.). In this vein, herein we focus on the development of biocompatible TTA-UC biomaterials for 3D cell cultures.

**Figure 1.**
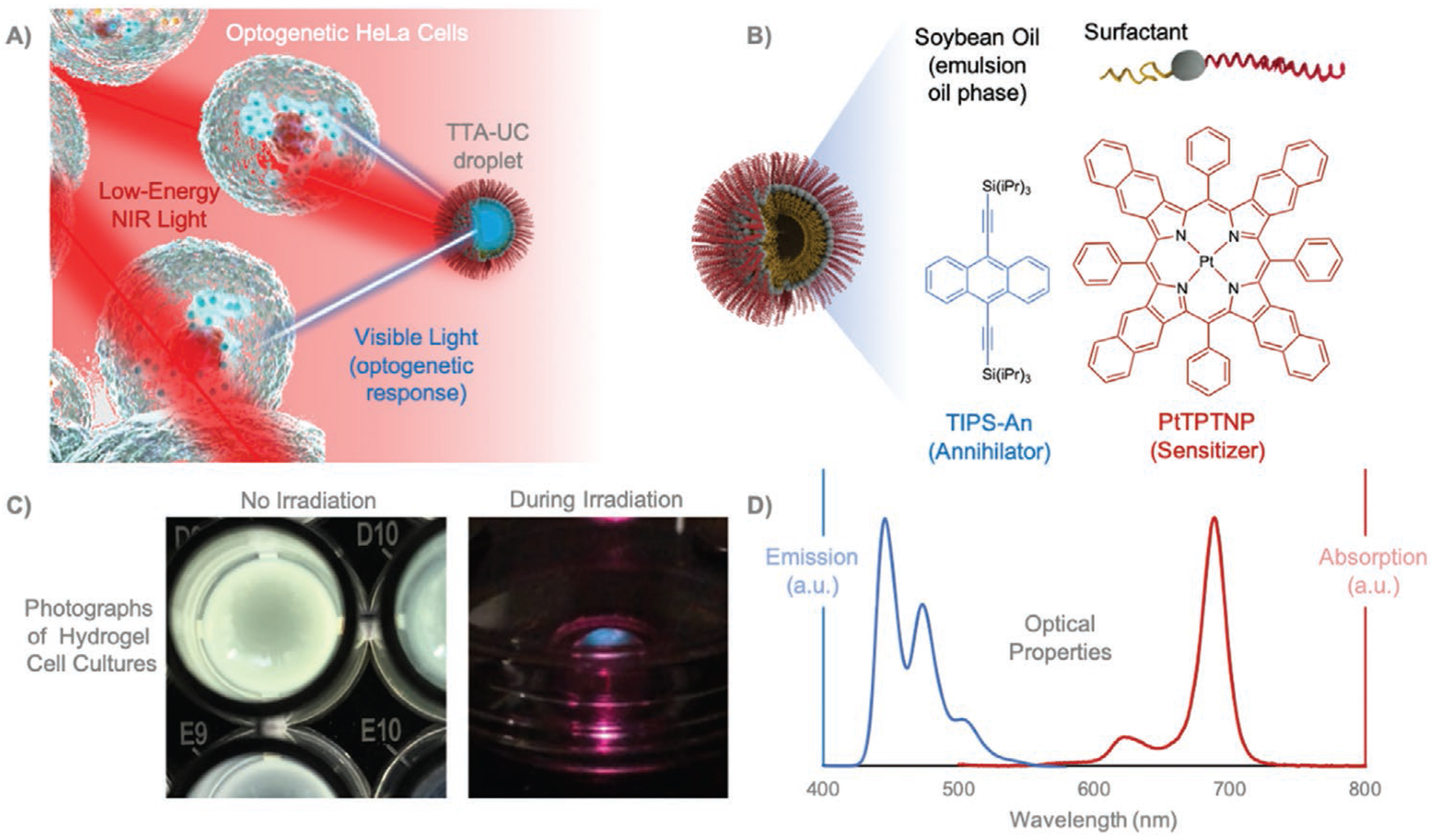
A) Representation of the concept of upconversion optogenetics within hydrogel biomaterials by NIR light penetration and local emission of blue light. B) Components comprising the upconversion micrometer-sized droplets: surfactant, reductant oil (soybean oil), sensitizer, and annihilator. C) Photograph of the hydrogels in microwells without and with NIR irradiation, where the irradiated gel emits blue light. D) Normalized absorption spectrum of the PtTPTNP sensitizer (red) and emission spectrum of the TIPS-An annihilator (blue).

While photon upconversion in biology has been mainly focused on using lanthanide-based inorganic nanoparticles,^[13,14]^ TTA-UC has received less attention in optogenetics^[15,22]^ mainly due to challenges associated with biocompatibility, sensitivity to oxygen, and insolubility of the organic chromophores in aqueous media.^[23,24]^ In most cases, nanoparticles containing TTA-UC chromophores have been used for drug delivery and imaging.^[25]^ Considering the challenges and significant potential applications of TTA-UC 3D biomaterials, we report the development of a materials platform of biocompatible hydrogels that are capable of absorbing near-IR (NIR) light (*λ* ≈ 700 nm) and emitting blue light (*λ* < 500 nm) within the 3D scaffold. As proof-of-principle, we investigate the ability to photochemically trigger a primary optogenetic response using HeLa cells expressing a variant of the optogenetic *Arabidopsis* photoreceptor Cryptochrome 2 (Cry2olig).^[26]^ The general concept is shown in Figure 1A,B, where the oil-in-water TTA-UC emulsion droplets are formed with a surfactant that encapsulates the sensitizer chromophore (NIR light absorber) and the annihilator (visible-light emitter) in soybean oil. The absorption and emission spectra of the two systems are also shown in Figure 1D to highlight the apparent anti-Stokes shift. Hydrogels are then formed, containing the HeLa cells expressing Cry2olig and the TTA-UC emulsion droplets under aerobic conditions in buffer. The resulting biomaterials are visibly opaque due to light scattering, but under blanket irradiation with NIR light, the hydrogels internally emit blue light (Figure 1C). The system is modular, as different combinations of TTA-UC chromophores and the hydrogel support network (e.g., synthetic or biopolymer derived hydrogels) can be readily modified.

## 2. Results and Discussion

In order to develop photoactive biomaterials that operate by TTA-UC, it is important to consider the characteristics of the multiexciton system together with the mechanism of upconversion, which has been extensively described in the literature (schematically detailed in Figure S1, Supporting Information).^[27,28]^ Previous studies have shown that TTA-UC hydrogels can be synthesized, but none have reported hydrogels containing cells dispersed within a 3D scaffold.^[15,29]^ In a recent eloquent study, Pluronic-based gels were simply used as a light source (NIR-to-blue light) placed above 2D cultures of hippocampal neurons to trigger optogenetic responses. However, we note that similar optogenetic responses can be observed in 2D cultures by direct irradiation with blue light.^[7]^ To obtain biocompatible, cell-laden TTA-UC hydrogels, we sought to integrate the sensitizer/annihilator pairs within the core of oil-in-water emulsion droplets^[24,25,28,30,31]^ that could be incorporated into cell-laden hydrogel matrices (e.g., fibrin gels). Several requirements must be met so that the photoinduced processes can efficiently lead to the desired optogenetic response(s). First, the TTA-UC chromophores must operate with NIR light input (*λ* ≈ 700 nm) and visible light output (*λ* < 500 nm) under aerobic conditions, where oxygen is generally detrimental to TTA-UC chromophores. Second, the oil-in-water emulsion droplets must be biocompatible and must be robust within the 3D biomaterial composite, that is, their contents must not be released into the environment. Finally, the biomaterials must contain NIR-silent/transparent components, but respond to the upconverted light within the hydrogel.

Considering the light input/output by TTA-UC to test an optogenetic response,^[7,15]^ we note that the majority of photoreceptors in engineered cells absorb blue light. In this study, we used Cry2olig,^[26]^ which is activated and undergoes oligomerization by photons in the range of 400–500 nm (peaking at 450 nm).^[32]^ Therefore, we examined two chromophores that have been recognized as efficient blue emitting annihilators: 9,10-((triisopropylsilyl)ethynyl)anthracene^[33]^ (TIPS-An; Figure 1B) and tetra-*tert*-butylperylene^[18]^ (TTBP; Figure S2, Supporting Information). These were paired with the triplet sensitizer palladium(II) meso-tetraphenyltetrabenzoporphyrin (PdTPTBP), which absorbs ≈640 nm light (Figure S3, Supporting Information) emitted by ordinary commercially available red diodes. While upconversion from red to blue light would give enhanced penetration of light, the need to efficiently sensitize within the biological transparency window would require longer wavelength of excitation (*λ* > 700 nm). There are only a limited number of NIR-absorbing sensitizers,^[33]^ but platinum(II) tetraphenyltetranaphthoporphyrin (PtTPTNP; Figure 1 and Figure S3, Supporting Information) can be paired with both TTBP^[18]^ and TIPS-An,^[33]^ exhibiting an apparent anti-Stokes shift of ≈1.0 eV. While both annihilators work well, we chose to focus on TIPS-An because it often outperforms TTBP in terms of upconversion efficiency,^[33,34]^ and it can be more readily prepared on a large scale.

Having identified suitable TTA-UC chromophore pairs for red-to-blue and NIR-to-blue upconversion, we set out to test the ability to achieve upconversion in biologically relevant systems. To date, most TTA-UC systems operate in deoxygenated organic solvents, which is antithetical to the environment that cultures of cells require.^[18]^ However, it is possible to encapsulate the chromophores within emulsions that contain a high boiling point non-polar organic solvent in the lipophilic core of the emulsion droplets (Figure 1B).^[30]^ These organic solvents (i.e., 1,2,4-trichlorobenzene, toluene, and 1,3,5-trimethylbenzene [TMB]) are necessary to prevent aggregation of the TTA-UC chromophores, but they can be toxic to living organisms. Nevertheless, it is possible to both solubilize and improve the oxidative stability of TTA-UC chromophores using reductive oils.^[35,36]^ Therefore, we tested a variety of emulsions for upconversion stability in the presence of oxygen and for cytotoxicity by varying the oil phase of the emulsions, including both natural soybean oil and D-limonene as antioxidant solvents^[35,36]^ and TMB as a control organic solvent. Finally, we tested the cytotoxicity of three types of non-ionic surfactants (Pluronic F127, Tween 80 and Triton X-100).

**Figure 2.**
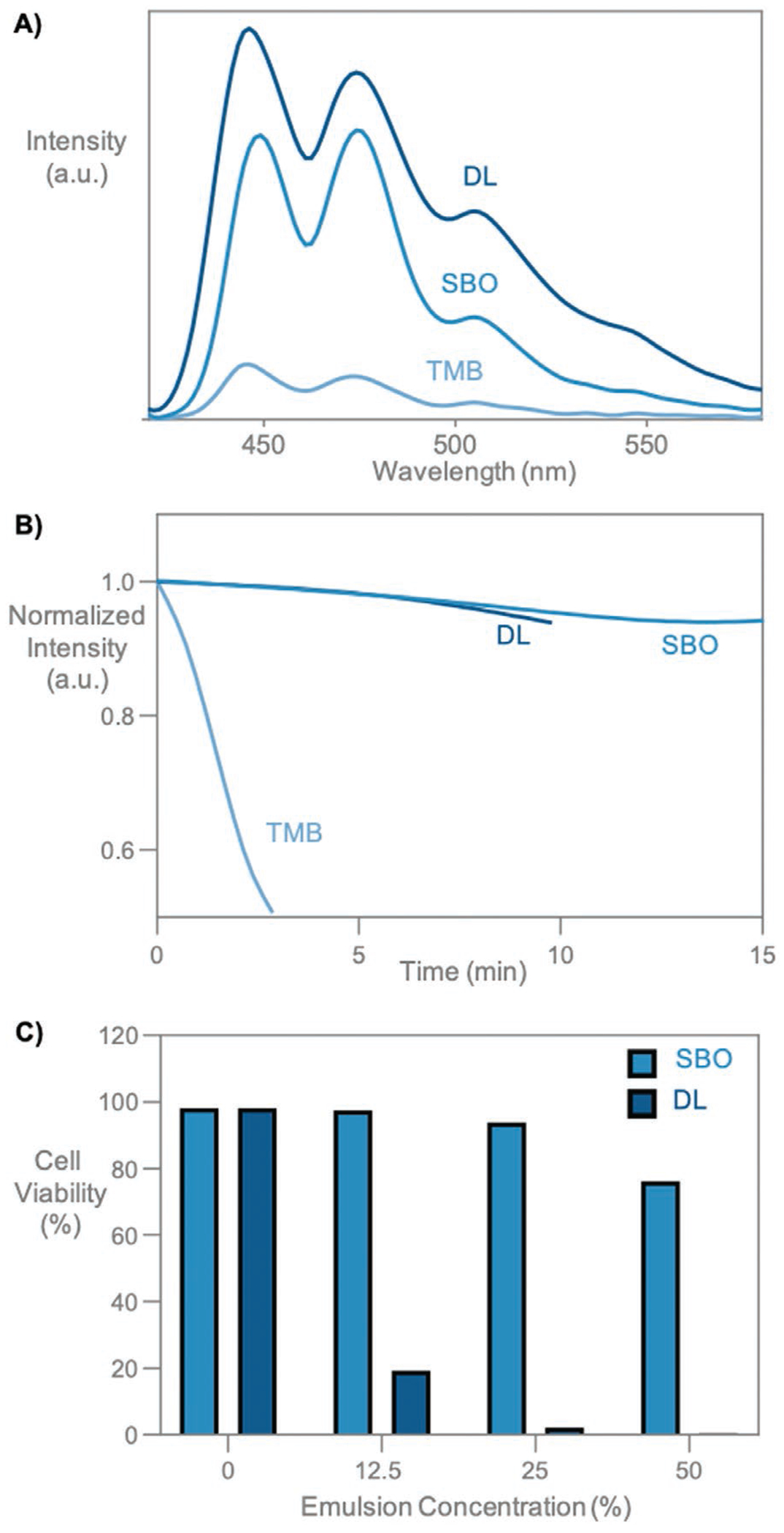
Photon upconversion in aerated conditions with TTA-UC emulsions and relative biocompatibility. A) Upconversion luminescence emission spectra of air saturated TTA-UC emulsions (TIPS-An:PdTPTBP) upon excitation with red light (637 nm) as measured after 1 min under constant irradiation using TMB, soybean oil (SBO) and D-limonene (DL), in the oil phase of the emulsion. B) Stability of upconversion emulsions over 15 min under constant irradiation. Plot shows intensity at various time intervals, relative to the initial intensity of the system. Intensities were monitored at the emission maxima (450 nm). C) Biocompatibility of the TTA-UC emulsions with D-limonene and soybean oil. Cell viability was measured at 1 h incubation with emulsion-containing media at different volume percent. D-limonene was found to be toxic to the cells even at low volumes while soybean oil was found to be remarkably less cytotoxic.

To evaluate the stability of the TTA-UC chromophores under irradiation in aerobic conditions, we used droplets based on Pluronic F127 surfactant and the TIPS-An/PdTPTBP annihilator/sensitizer pair. The emulsions were exposed to continuous irradiation with a 637 nm laser for 15 min (76 mW mm^−2^) and a spectral detector was used to measure luminescence of visible light at one-min intervals (spectral range 400–580 nm). Figure 2A shows the photoluminescence (PL) emission spectrum of TIPS-An from the TTA-UC emulsions. We found that both soybean oil and D-limonene as the oil phase of the emulsion qualitatively exhibited higher upconversion intensity than TMB (Figure 2A, see details in the Supporting Information) and the luminescence remained constant for the entire duration of the experiment (15 min; Figure 2B). The luminescence of droplets containing TMB was further reduced to 50% of its initial luminescence within 4 min of irradiation (Figure 2B). Since both soybean oil and D-limonene provided higher stability to the chromophores than TMB, we examined the biocompatibility of these two emulsions. Upon exposing cells to the upconversion emulsions for 1 h using Pluronic F127 surfactant, with and without chromophores, and at different concentrations, the D-limonene emulsion droplets were found to be toxic to the cells. Interestingly, cells exposed to low concentrations of D-limonene droplets without chromophores had low viability, while the soybean oil droplets were found to be biocompatible, with and without chromophores, at high concentrations (Figure 2C and Figure S4A, Supporting Information). Among the three surfactants that were tested (Pluronic F127, Tween 80 and Triton X-100), only Pluronic F127 was found to be biocompatible and nontoxic to the cells at high concentrations (Figure S4B, Supporting Information). Thus, we concluded that using soybean oil in the core of the droplets and Pluronic F127 (10 wt%) as a surfactant were biocompatible, and resulted in efficient upconversion in an aqueous and air saturated environment. We also found that the size of the emulsion droplets was important. While other work has focused on nanoscale micelles,^[30]^ we found that increasing the size of the emulsion droplets is necessary to prevent their release from the hydrogel matrix into the environment (Figures S5 and S6, Supporting Information). Microscale droplets, with a mean diameter of ≈1.9 μm, successfully entrap within the hydrogel matrix without leaching.

**Figure 3.**
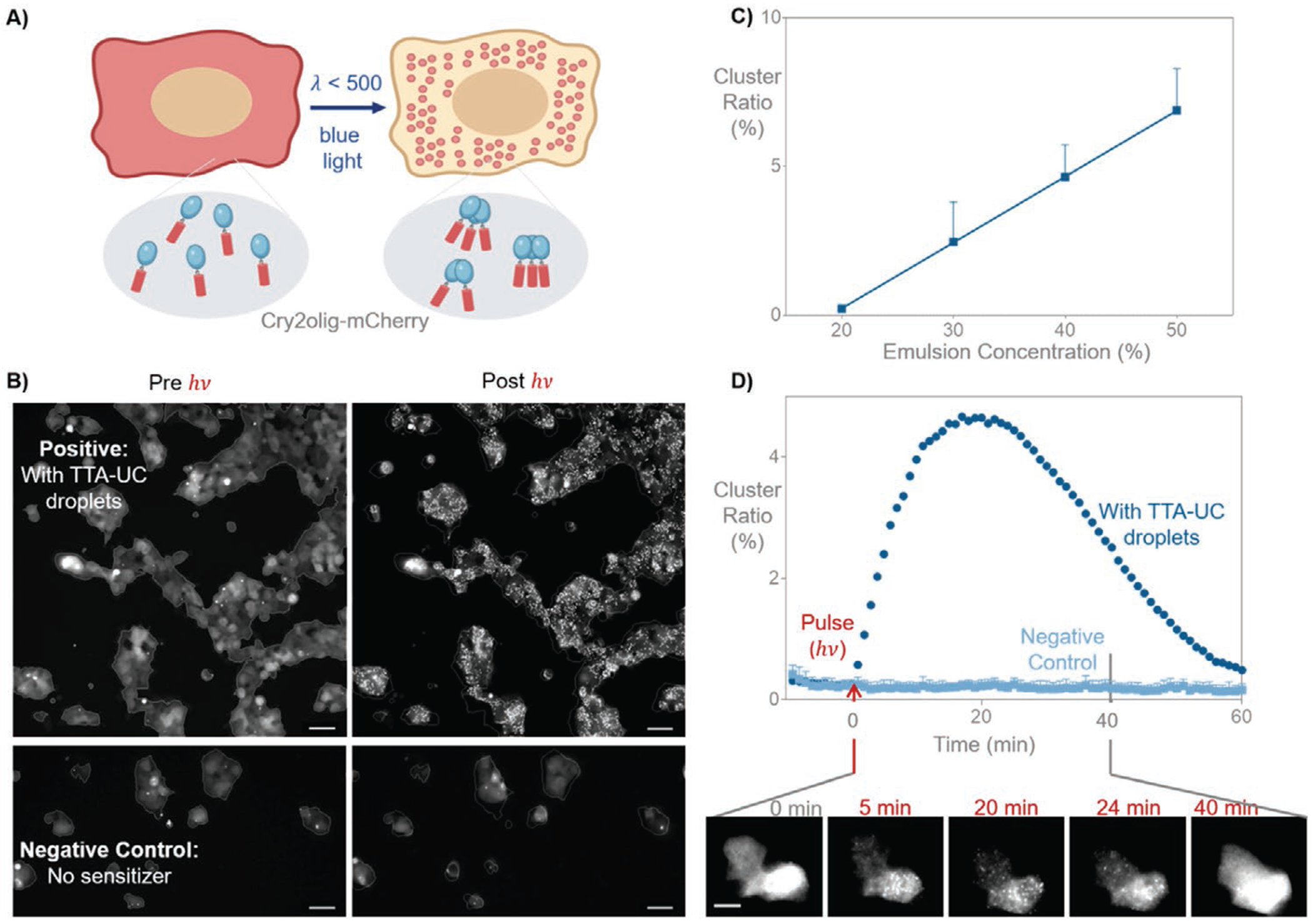
Upconversion-mediated dynamic optogenetic response in 2D. A) Schematic describing of the model optogenetic system; HeLa cells express Cry2olig-mChy that oligomerizes and forms clusters in response to blue light. B) Fluorescence microscopy images of cells expressing Cry2olig-mChy clearly exhibit oligomerization mediated by the TIPS-An:PdTPTBP upconversion emulsion, 10 min post stimulation with red light (1 s, 637 nm LED at 11 mW). Cells exposed to control emulsion (TIPS-An only) did not cluster in response to red light (637 nm LED at 11–55 mW). Scale bars, 50 μm. C) Correlation between the number of clusters observed and the concentration of the upconversion emulsion in the cell media (mean ± s.d., *n* = 12 sites per concentration). Quantification of the cluster ratio in cells, calculated by dividing the total cluster intensity by the total fluorescence of the whole cell (see Supporting Information for details). D) Plot of time-lapse results showing changes in cluster ratio (mean, *n* = 12 sites per condition) and representative images (scale bar, 25 μm), demonstrating reversibility of the optogenetic process.

Having identified appropriate non-cytotoxic TTA-UC emulsions, we tested whether the energy output of the TTA-UC process from the emulsions would suffice to activate photoreceptors in optogenetic cells in 2D cell cultures,^[32]^ as an initial step before introducing the hydrogel matrix. As a model system, we used the Cry2olig, which induces rapid and reversible protein oligomerization in response to blue light (Figure 3A).^[26]^ For the 2D experiments, HeLa cells were engineered to express Cry2olig fused to mCherry construct (Cry2olig-mChy) and were cultured as monolayer on glass-bottom plates one day prior to photostimulation. Using a light emitting diode (LED) with a pulse of blue light (*λ_ex_* = 488 nm, 1 s), we confirmed the formation of clusters of Cry2olig in response to blue light (Figure S7A, Supporting Information). Next, the TTA-UC emulsion (TIPS-An:PdTPTBP) or control emulsion (TIPS-An only) were mixed into the cell media at various concentrations (10 to 50% volume). For upconversion-mediated excitation, we irradiated the cells with a pulse of red LED (*λ_ex_* = 637 nm, 1 s, 11 to 55 mW). Fluorescence microscopy imaging was used to track the formation of clusters of the Cry2olig-mChy construct before and after irradiation. Before light stimulation, most of the Cry2olig-mChy signal appeared to be distributed throughout the cell cytoplasm (Figure 3B, pre-*hν*). Upon red light stimulation under aerobic conditions in the presence of the upconversion emulsion, we clearly observed cluster formation of Cry2olig-mChy (Figure 3B top, post-*hν*). We found a linear correlation between the concentration of the upconversion emulations in the cell media and the number of clusters formed, with a threshold of 20% volume for cluster formation (Figure 3C). The control experiment without the inclusion of the light-absorbing sensitizer in the emulsion shows no cluster formation (Figure 3B, negative control, post-*hν*). Furthermore, Figure 3D shows the reversibility of the upconversion-mediated optogenetic process, as the number of clusters increased following the light pulse and then decreased over time, as expected from the Cry2olig-mChy construct.^[26]^ Of note, cells that were exposed to the TTA-UC emulsions under irradiation remained vital for the duration of the experiment (70 min) for all TTA-UC emulsion concentrations tested. Next, we carried out similar experiments, but using the NIR-to-blue upconversion emulsion loaded with PtTPTNP as the sensitizer (TIPS-An:PtTPTNP, *λ_ex_* = 710 nm). Not surprisingly, similar upconversion-mediated clustering in response to NIR light stimulation was observed (Figure S7B, Supporting Information), while no clustering was detected with control emulsions (TIPS-An only; Figure S7C, Supporting Information).

**Figure 4.**
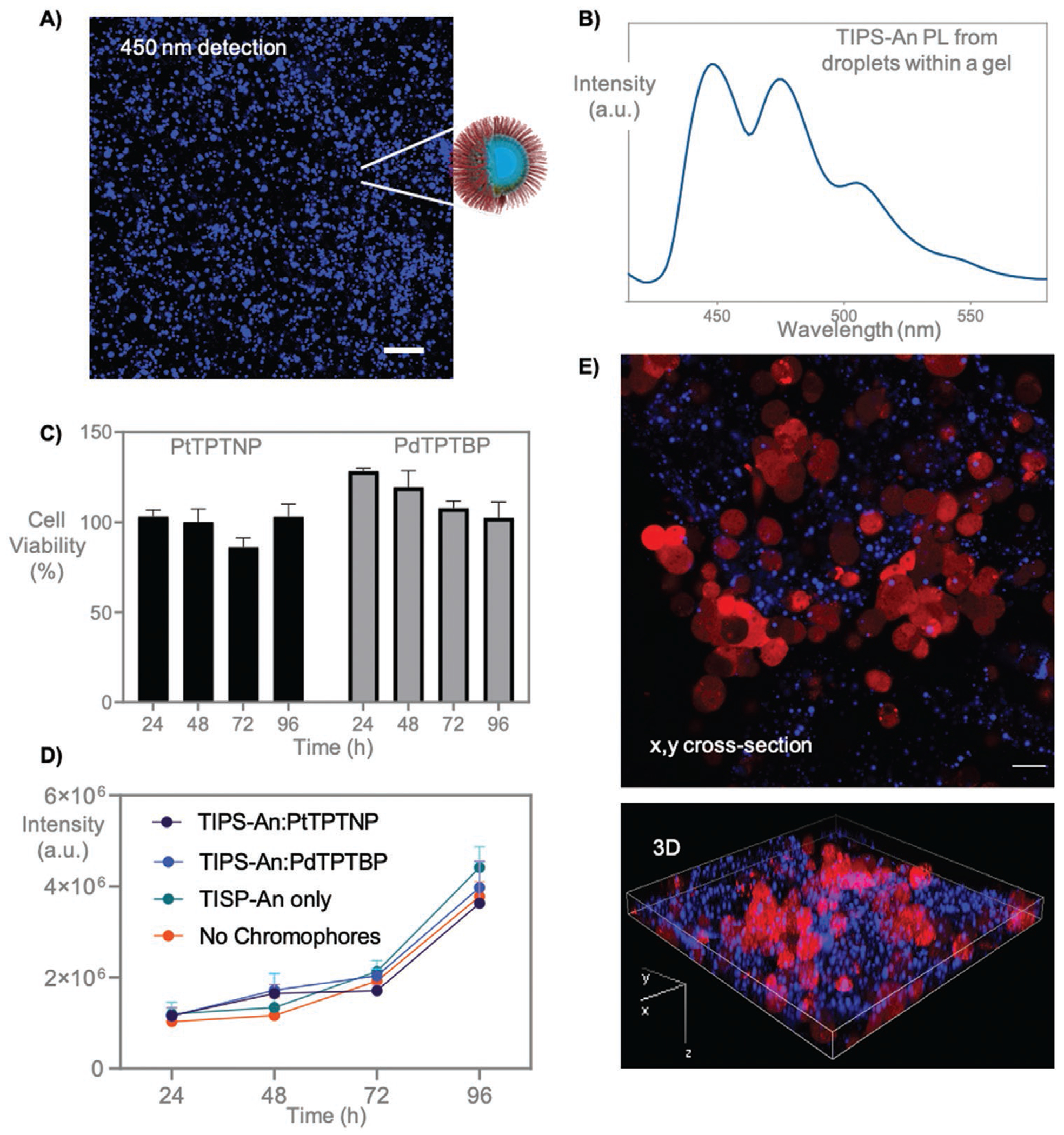
Characterization of cell-laden TTA-UC hydrogels. A) Confocal image of fibrin hydrogels with TTA-UC droplets, imaged by exciting with a red laser (637 nm) and detecting the upconverted emission of blue light (450 nm). Scale bar, 25 μm. B) TTA-UC emission spectrum of the droplets imaged in A. C) Viability of cells encapsulated in the TTA-UC hydrogels including TIPS-An:PtTPTNP or TIPS-An:PdTPTBP emulsions, at various time points post encapsulation, normalized to hydrogels without emulsions (mean ± SEM, *n* = 9). D) Viability of cells encapsulated in the TTA-UC hydrogels at various time points post encapsulation. The emulsions contain TIPS-An:PtTPTNP, TIPS-An:PdTPTBP, TIPS-An (no sensitizer) or emulsions without chromophores (mean ± SEM, *n* = 9). The signal intensity increases over time due to proliferation of the cells within the hydrogels. E) Confocal images of cells encapsulated in the TTA-UC hydrogels, highlighting the distribution of the droplets and cells within the 3D matrix: HeLa cells (red, excitation 561 nm) and TTA-UC droplets (blue, excitation 405 nm). Top: cross-sectional image (*x*,*y*-plane, scale bar 25 μm.); Bottom: 3D rendering (x, y, z).

Encouraged that the TTA-UC emulsions triggered an optogenetic response in a monolayer of cells (2D), we next sought to test the performance of the upconversion emulsion droplets dispersed within a fibrin gel (3D), a commonly used hydrogel matrix.^[37]^ The cell-loaded TTA-UC hydrogels were prepared by adding the TTA-UC emulsion (5% by volume) to the cellcontaining fibrinogen solution before the biopolymer was crosslinked with the Thrombin enzyme (Figure S8A, Supporting Information). From the resulting free-standing gel, blue light emission can be observed with the naked eye upon stimulation with red light (Figure 1C and Figure S8B, Supporting Information). Moreover, Figure 4A shows the confocal microscopy images of the emitted light from the widely dispersed droplets within TTA-UC hydrogels based on TIPS-An:PdTPTBP (see Figure S8C, Supporting Information, for other detection windows). From these gels, the PL emission spectrum shown in Figure 4B matches that of TIPS-An. The stability of the TTA-UC signal in the hydrogels (TIPS-An:PdTPTBP) was tested by continuous irradiation from a red laser (*λ_ex_* = 637 nm, 76 mW mm^−2^), and no loss of signal was observed after 15 min (Figure S9, Supporting Information), similar to the signal stability in 2D (Figure 2B). The 3D resolution of the TTA-UC hydrogels is fundamentally defined by the penetration depth of red (*λ* ≈ 640 nm) and NIR (*λ* > 700 nm) light through the cell-laden hydrogels. It is well known that the scattering and absorption coefficients of biological tissues are strongly wavelength dependent.^[38]^ While blue light offers limited penetration through tissue, the advantage of using red and NIR light for excitation of a system is that these wavelengths lie within the biological optical window (640–950 nm). For example, for light penetration through human skin, red and NIR light is extinguished ≈5 mm beneath the surface of the skin, whereas blue light penetrates ≈1 mm into tissue.^[20]^ Given that the hydrogels are opaque and scatter blue light, the penetration of 640 nm light through the upconverting hydrogel was qualitatively characterized. A sample with dimensions of 1 cm × 1 cm × 2.5 cm was synthesized in a cuvette and irradiated through the long axis (Figure S10, Supporting Information) at ambient conditions. In this sample, upconverted blue light was visible and photographs show scattered blue light emanating throughout the opaque gel. Next, cell viability assays show that the hydrogels are biocompatible for at least 4 days post-encapsulation using both TIPS-An:PdTPTBP and TIPS-An:PtTPTNP emulsion droplets within the hydrogel (Figure 4C). Moreover, the hydrogel supports cell proliferation as shown by the increase in signal over time (Figure 4D). Furthermore, microscopy images show the coexistence of the cells and emulsion droplets dispersed within the upconversion hydrogel for at least four days (Figure 4E and Figure S11, Supporting Information).

**Figure 5.**
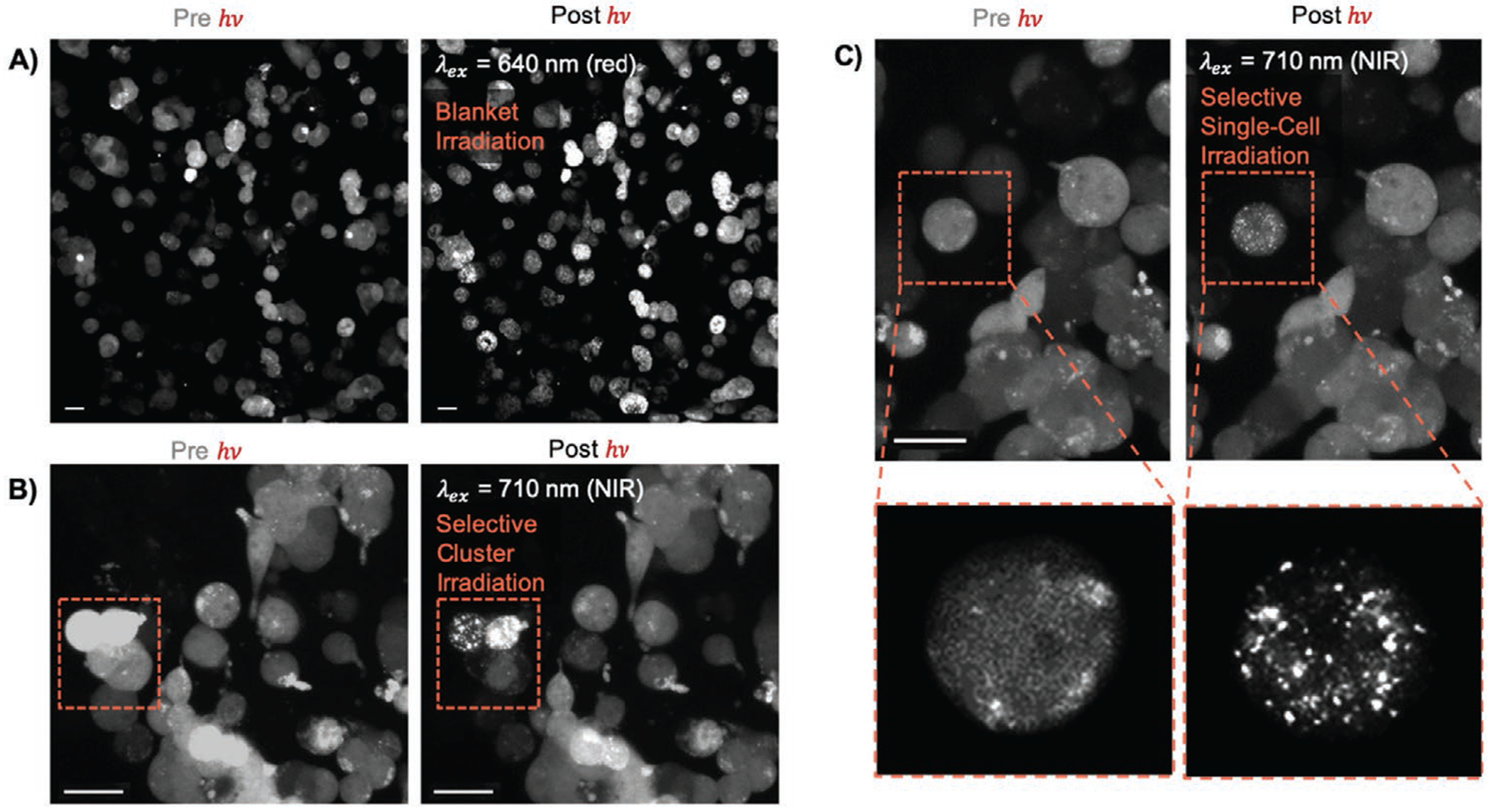
Optogenetic responses from HeLa cells down to single-cell activation. Representative max intensity projections of confocal images of the HeLa cells encapsulated within the TTA-UC biomaterial two days post encapsulation: before irradiation and 10 min after irradiation with: A) 637 nm laser excitation, showing Cry2olig-mChy photoreceptors oligomerize in response to the upconverted red-to-blue light from the TIPS-An:PdPTPBP system. B) 710 nm scanning laser excitation, focused on a small cluster of cells (in red frame), showing response of cells to NIR-to-blue light from the TIPS-An:PtTPTNP system. C) Representative experiment demonstrating single cell clustering in response to 710 nm excitation within TIPS-An:PtTPTNP TTA-UC hydrogel. Scale bars 25 μm.

In order to test the ability to stimulate the optogenetic cells inside the cell-laden TTA-UC biomaterial using red and NIR light, we turn to investigate the 3D optogenetic response from the engineered HeLa cells. First, HeLa cells expressing Cry2olig-mChy were encapsulated into the fibrin hydrogels containing 5% (by volume) of the TTA-UC emulsion (TIPS-An:PdTPTBP or TIPS-An:PtTPTNP) or control emulsions (TIPS-An only). Two days post encapsulation in the hydrogel, the cells were stimulated with a red or NIR laser and imaged by confocal microscopy (see details in the Supporting Information). As evidenced in Figure 5A, upon blanket irradiation with red-light (*λ_ex_* = 637 nm, 7.6 mW), we clearly observed the formation of Cry2olig clusters—the primary optogenetic response—while the control hydrogels without the sensitizer did not show clustering (Figure S12A, Supporting Information). Additionally, we were able to observe the formation of clusters upon stimulating the cell-laden TTA-UC gels with a NIR scanning laser (*λ_ex_* = 710 nm, 63 mW, see Figure S13, Supporting Information).

Given the homogenous optogenetic response of the cells in 3D, we posit that local irradiation could elicit an optogenetic response from a select group of cells. The most widely studied methods to trigger/control cellular processes can only be done at steady state through chemical cues in the culture media^[39,40]^ or by modifications to their immediate environment in 2D^[41–45]^ and 3D.^[46]^ Thus, it is of utmost interest to develop technologies to achieve spatiotemporal control of cellular behavior, down to single-cell resolution.^[47,48]^ By focusing the microscope NIR laser on a specific group of cells, we were able to trigger the TTA-UC process in their vicinity with precision, which then led to the clustering of Cry2olig. Figure 5B shows confocal images of the cells dispersed in the 3D matrix before focused irradiation (pre *hν*) and after NIR light exposure only in specific sites of irradiation (post *hν*, the irradiated region is highlighted in the images). It is remarkable to observe the lack of Cry2olig cluster formation from cells within the vicinity of focused irradiation of the TTA-UC droplets. This experiment truly highlights the ability to use long-wavelength light to trigger cellular processes with spatiotemporal control in 3D. Moreover, we were able to trigger the optogenetic response of a single cell (Figure 5C). To date, such level of specificity in a 3D matrix is unprecedented. We postulate that this class of biomaterials will open up new avenues of exploration of cell signaling mechanisms, 3D printing, drug delivery, and tissue engineering, among others.^[49,50]^ It is noteworthy that these experiments were reproducible over several days and we were able to image and stimulate cells in the hydrogels on days 0–3 post encapsulation (see Figures S13 and S14, Supporting Information). The control hydrogels without the sensitizer did not show clustering of the proteins within a region on interest under NIR light irradiation (Figure S12B, Supporting Information).

## 3. Conclusions

Here we developed a class of 3D biomaterials that are capable of absorbing red/NIR light, which is upconverted to blue light by TTA-UC. These photon upconversion hydrogel biomaterials were found to photochemically induce optogenetic responses in cell-laden hydrogels enabled by TTA-UC. With these systems, it is now possible to photostimulate cells within a 3D biomaterial, overcoming the fundamental limitations imposed by direct irradiation with blue light. Adapting upconversion chromophores to function under ambient conditions has been an obstacle for interfacing these systems with living organisms as most systems are limited to deoxygenated organic solvents. However, we were able to overcome this challenge by incorporating the upconversion chromophores into emulsions containing soybean oil, a reductive solvent that consumes any generated singlet oxygen by TTA-UC, thus increasing the photostability without compromising biocompatibility and cytotoxicity. Furthermore, the rather large microdroplets of the emulsion prevented leaching from the hydrogel to the environment. Overall, the optimized composition of the 3D TTA-UC biomaterials led to the photochemical activation of optogenetic HeLa cells within 3D biomaterials. Moreover, we were able to observe the primary optogenetic response of a single-cell within a cell-laden hydrogel, exerting spatiotemporal control using a scanning laser. The successful development of 3D biomaterials is especially significant because the methodology introduces a new way to initiate photochemical processes of optogenetically engineered cells using low-energy light, with unprecedented precision.

## Supplementary Material

Supplementary Material Supporting Information is available from the Wiley Online Library or from the author.

## Data Availability

Research data are not shared.
