# Supplementary material for: Photon Upconversion Hydrogels for 3D Optogenetics
Source: Adv Funct Mater. Author manuscript; Available in PMC 2026 Aug 18. (PMC13480420; doi:10.1002/adfm.202010907)
Supplement: Supplementary file 1 [file NIHMS2200809-supplement-Supplementary_Material.pdf]

© 2021 Wiley-VCH GmbH

ADVANCED FUNCTIONAL MATERIALS

Supporting Information

for *Adv. Funct. Mater.,* DOI: 10.1002/adfm.202010907

Photon Upconversion Hydrogels for 3D Optogenetics

*Rinat Meir, Tal Hirschhorn, Sungsoo Kim, Kealan J. Fallon, Emily M. Churchill, Dino Wu, Hee Won Yang, Brent R. Stockwell, and Luis M. Campos**

Supporting Information

Photon Upconversion Hydrogels for 3D Optogenetics

*Rinat Meir, Tal Hirschhorn, Sungsoo Kim, Kealan J. Fallon, Emily M. Churchill, Dino Wu, Hee Won Yang, Brent R. Stockwell, and Luis M. Campos**

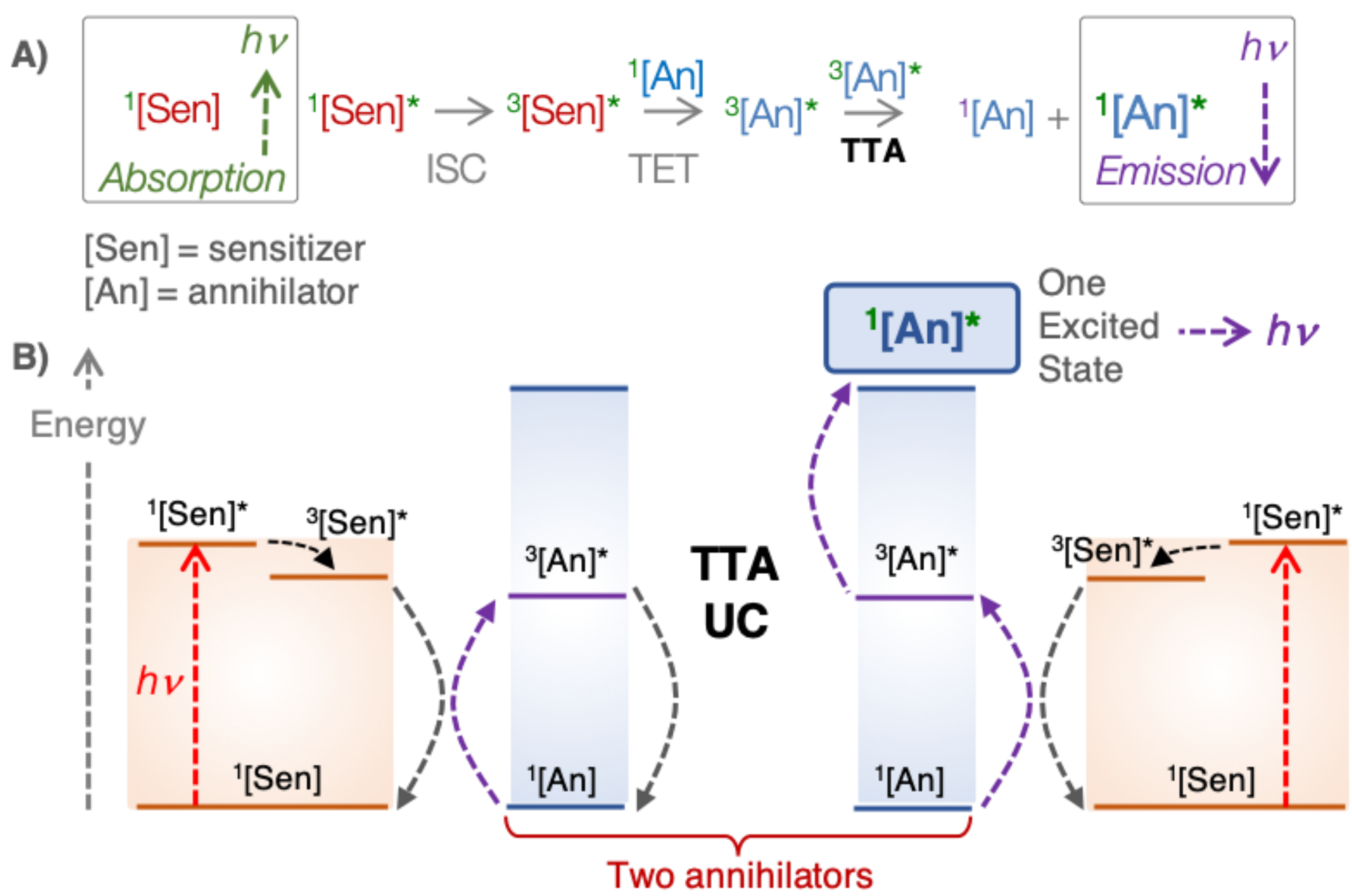

Figure S1. The process of triplet-triplet annihilation upconversion (TTA-UC) involves two chemical species, a sensitizer (Sen) and an annihilator (An). We use the notation ^{m}[Species]* to represent the multiplicity (m), which is either singlet (1) or triplet (3), and the excited state (*) or ground state (no asterisk). The net process is shown in (A), whilst the corresponding Jablonski

diagram is shown in (B). The ground-state sensitizer ^{1}[Sen] is excited with low-energy light ($h\nu$); the singlet exciton ^{1}[Sen]* undergoes fast intersystem crossing (ISC) to its triplet state ^{3}[Sen]*, which then undergoes efficient triplet energy transfer (TET) to the annihilator ^{3}[An]*; and finally, two annihilator triplet excitons (^{3}[An]*) can undergo triplet-triplet annihilation to yield one annihilator in the ground state ^{1}[An] and other in the singlet state ^{1}[An]* which then decays radiatively via fluorescence to emit a photon of high-energy light ($h\nu$). (Adapted from reference [1]).

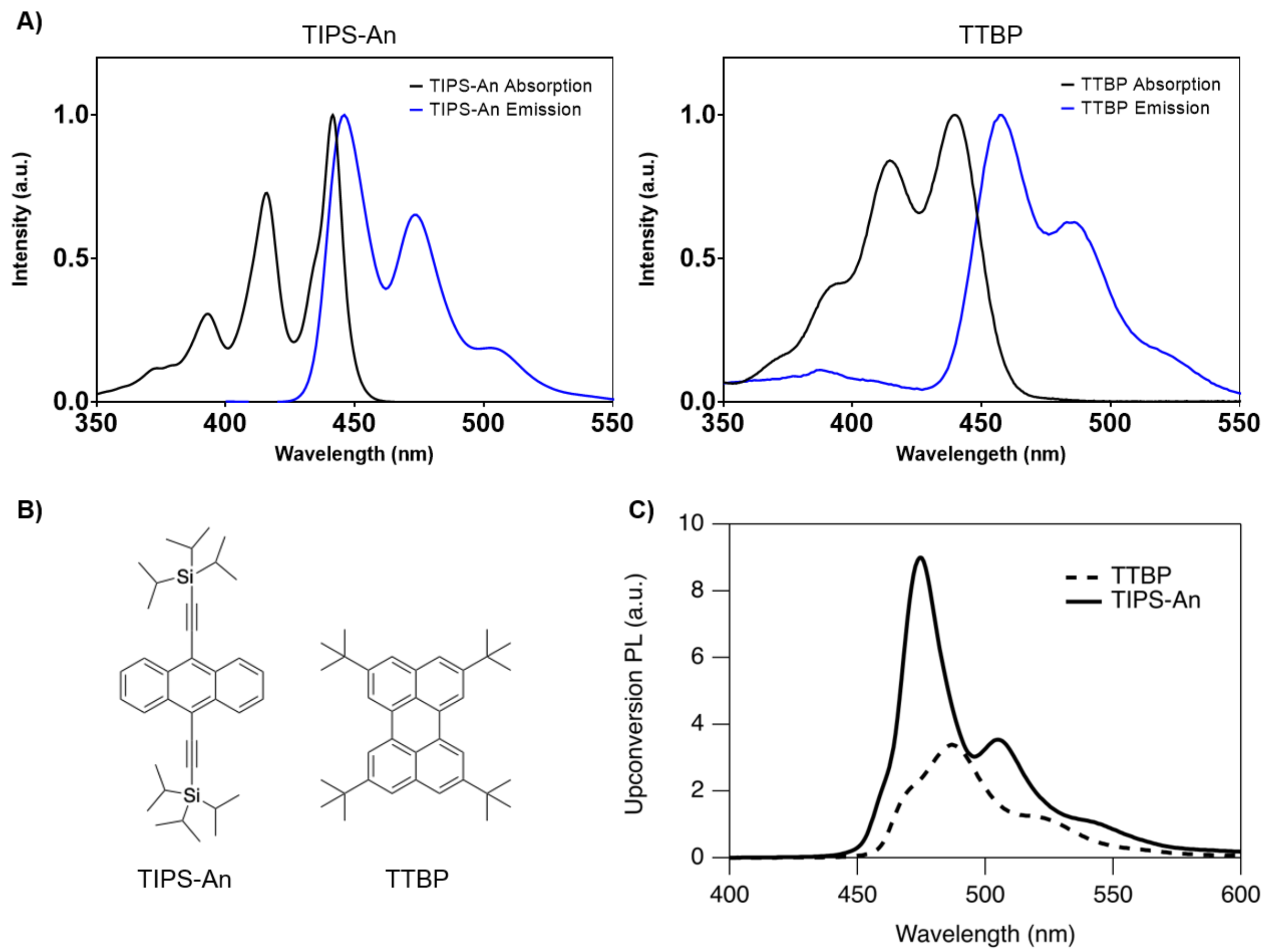

Figure S2. Annihilators used in this study. A) Absorption (black) and emission (blue) spectra of TIPS-An and TTBP taken in dilute toluene. **B)** Chemical structures of TIPS-An and TTBP. **C)** Relative upconversion photoluminescence of TIPS-An and TTBP demonstrating that TIPS-An shows more intense emission in the 450-500 nm range, compared to TTBP.

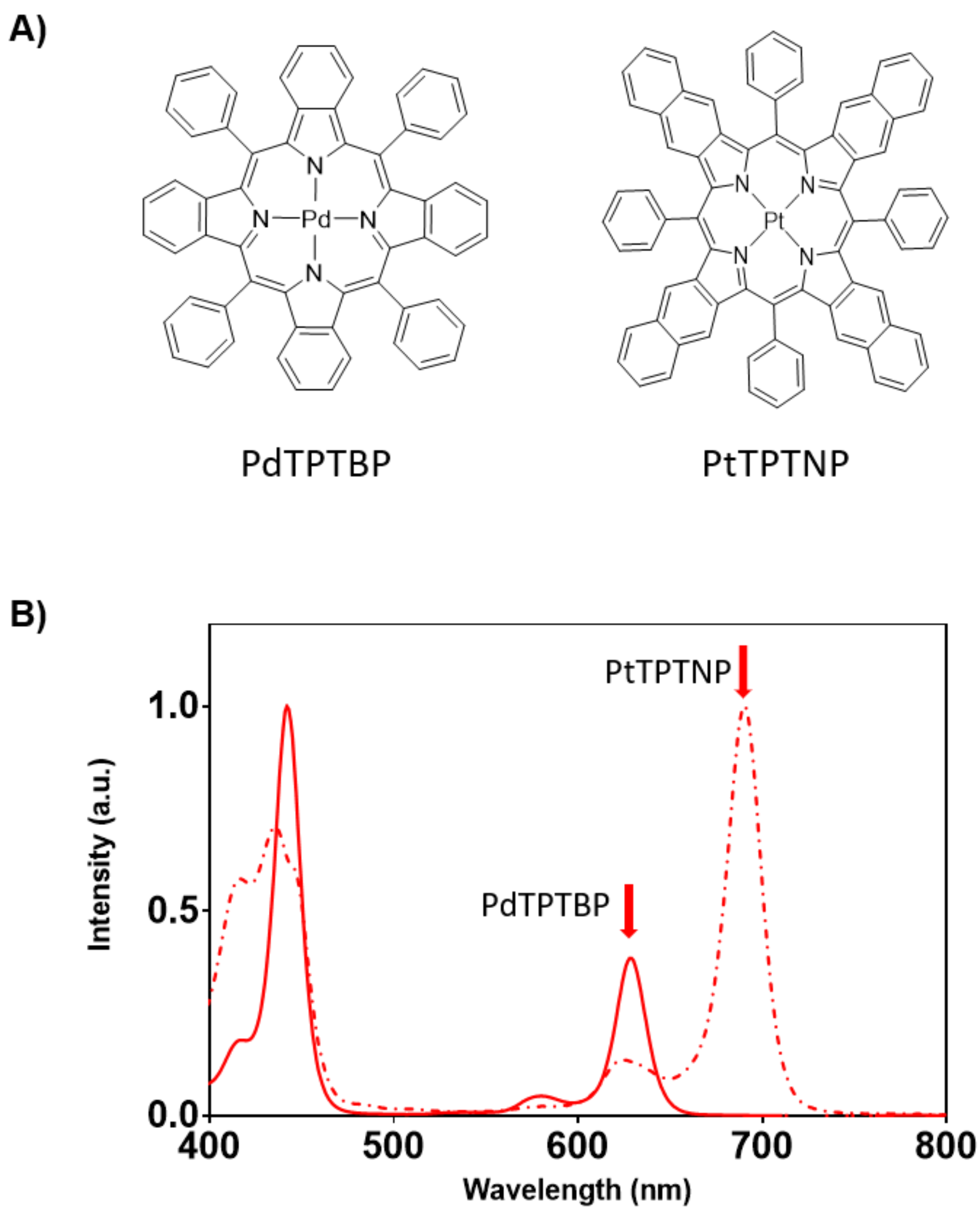

Figure S3. Sensitizers used in this study. A) The chemical structures of the two sensitizers used in this study: PdPTPBP and PtTPTNP. **B)** Normalized steady state absorption spectrum measured in dilute toluene, showing the intense Q-bands of the porphyrins: approximately 640 nm for PdTPTBP and 700 nm for PtTPTNP.

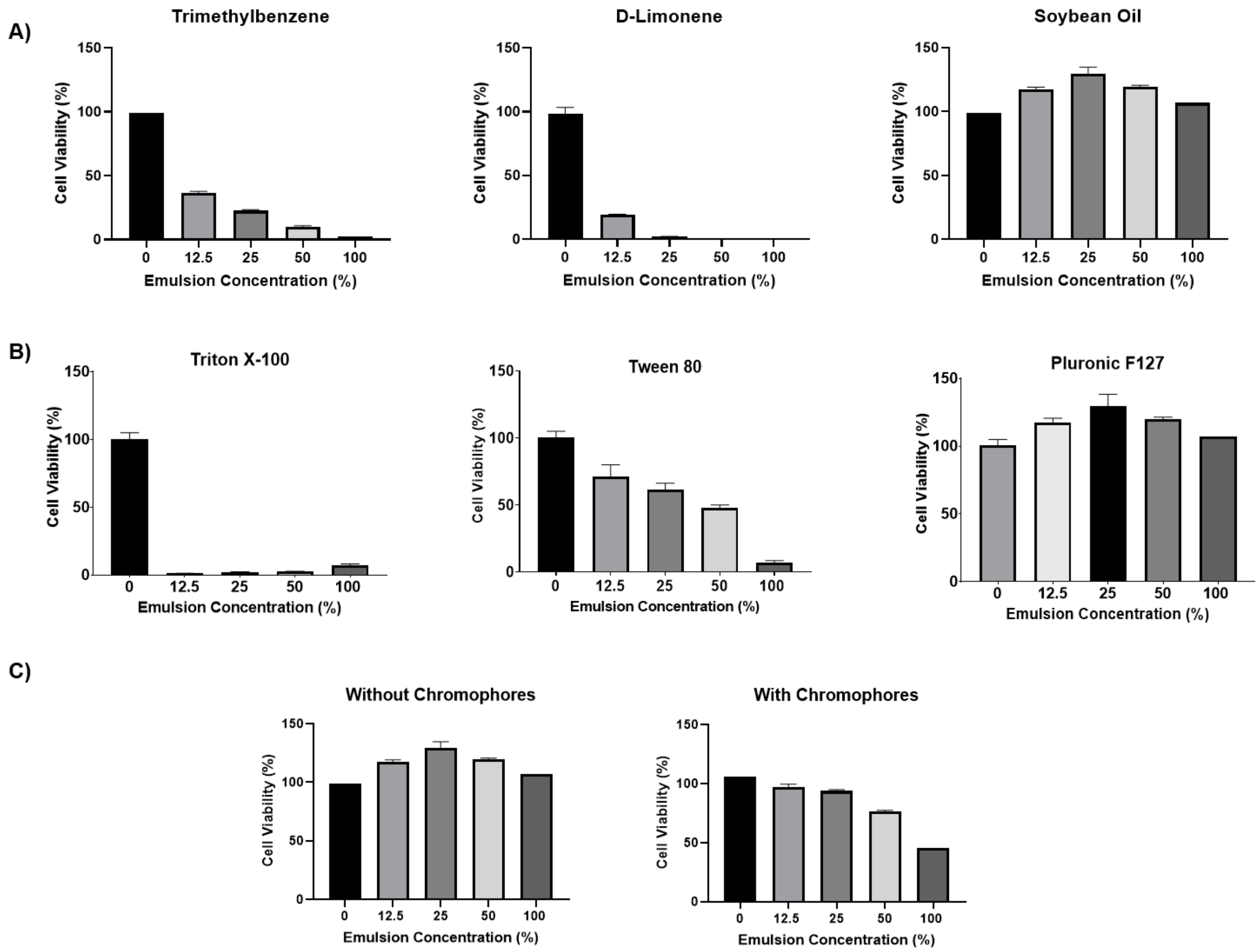

Figure S4. Biocompatibility of emulsions with different reductant oils. CellTiter-Glo® Luminescent Cell Viability Assay was used to determine the precent of viable HeLa cells in culture following 1 hour incubation with upconversion emulsions in different concentrations. In each data point, measured viability values were normalized to the measured viability of cells that were not incubated with emulsion droplets (100% viability). **A)** HeLa cell viability as a function of organic solvent contained within the droplets. **B)** HeLa cell viability as a function of emulsion surfactants. **C)** HeLa cell viability upon incubation with droplets containing soybean oil, with and without the photon upconversion chromophores.

A)

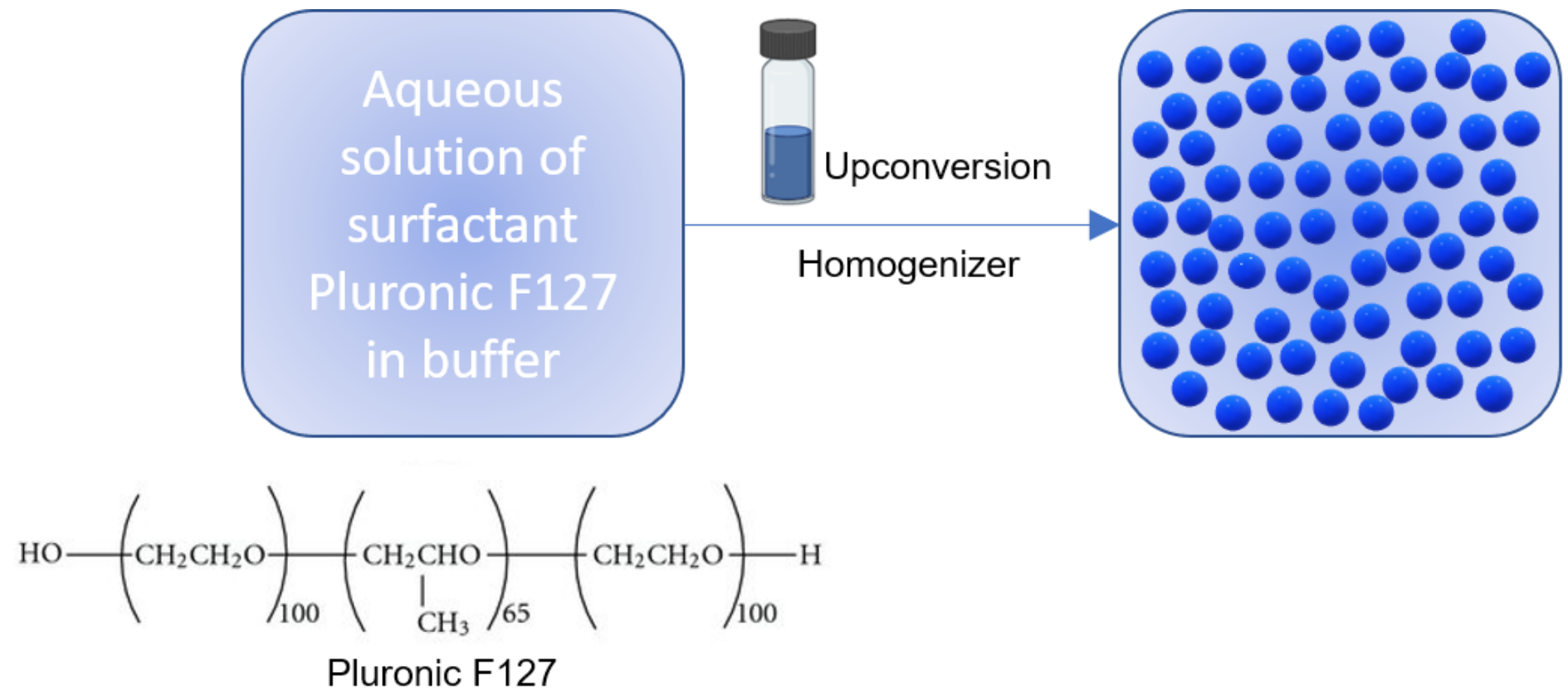

B)

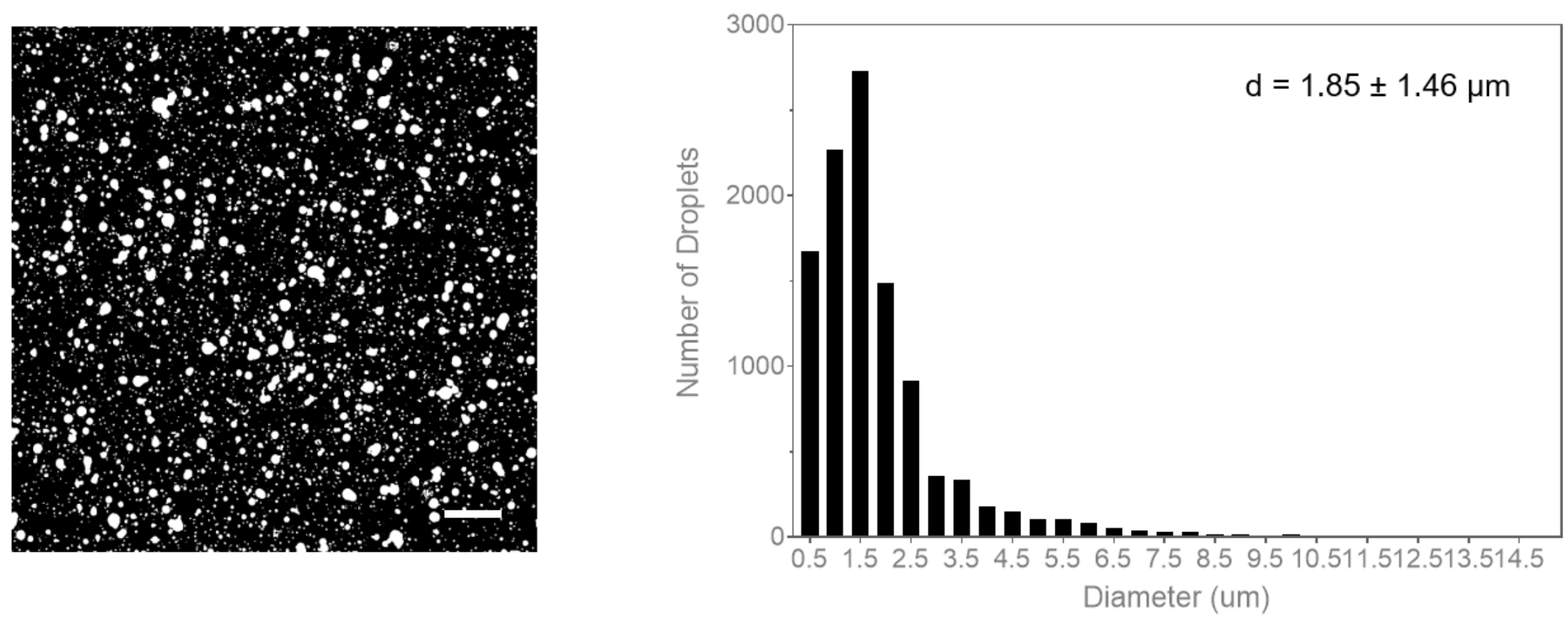

Figure S5. Synthesis and characterization of upconversion emulsion droplets. A) An oil-in-water emulsion is generated by adding a mixture of an oil containing the annihilator and sensitizer chromophores to a buffer solution containing Pluronic F127 (10 wt%). **B)** Left: Maximum intensity projection of the emulsion droplets as imaged by confocal microscopy. Scale bar, 25 µm. Right: Size distribution of the droplets was measured by imaging processing, using particle analysis. Diameter was determined as the maximum Feret diameter of each droplet. Diameter equals to 1.85 ± 1.46 µm (mean ± s.d.).

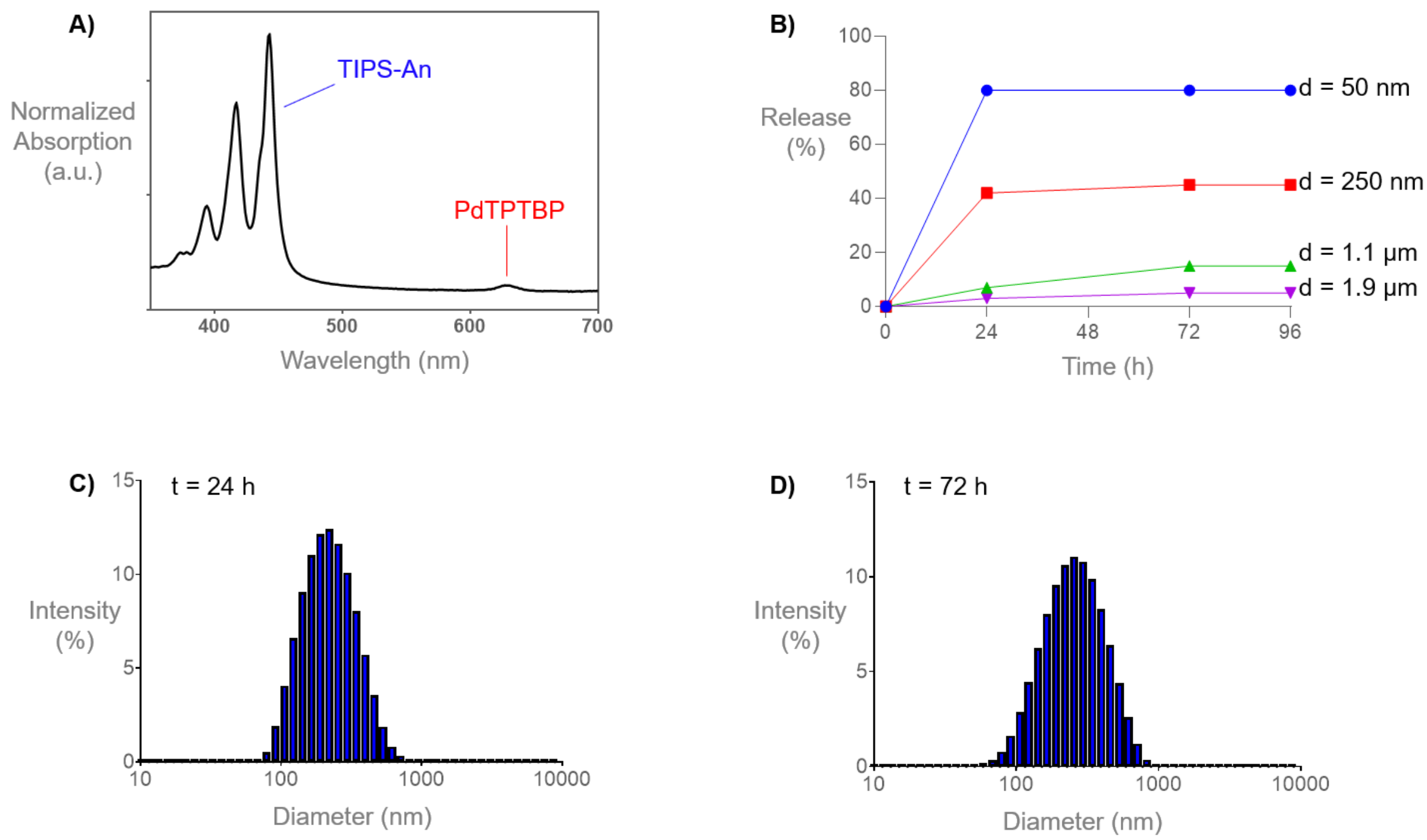

Figure S6. Assessment of droplet size dependency on the release of the droplets from the hydrogel. To show that increasing the size of the droplets enables to prevent their release from the hydrogels over time, we characterized the content of the supernatant buffer over time. First, hydrogels were formed using droplets (5% by volume) of different sizes (droplets mean diameter 50 nm, 250 nm, 1.1 µm and 1.9 µm). The hydrogels were covered with PBS buffer and monitored for four days. The resulting supernatant buffer was removed every 24 h from the hydrogels and characterized by using dynamic light scattering (DLS) and UV-Vis absorption spectroscopy. **A)** UV-vis absorption spectra of the supernatant buffer collected from the hydrogels 24 h post formation (droplet mean diameter 250 nm). The range of absorption wavelength 400 - 450 nm is characteristic of the annihilator TIPS-An, and the peak at 627 nm is characteristic of the sensitizer PdTPTBP. The result suggests that the droplets that are released to the buffer contain both the annihilator and the sensitizer. **B)** To quantify the relative release of the droplets from the hydrogels to the surrounding media, solution UV-vis absorption spectroscopy was employed to detect the relative concentration of chromophores in the supernatant of the hydrogels at different time points. Data is normalized to the concentration of chromophores in the control groups that were prepared without the addition of the hydrogel crosslinker to simulate a complete release of the droplets from the hydrogels to the supernatant (corresponding to 100% release of droplets). Data is presented as mean (n=3), “d” is the mean diameter of the droplets used to form the hydrogels for each group. For the smallest droplets (mean diameter 50 nm), 80% of the droplets were released from the hydrogels within 24 hours. Increasing the size of the emulsion droplets significantly decreases the release of the droplets from the hydrogel. **C,D)** Supernatant buffer was further characterized by using dynamic light scattering (DLS), to confirm the presence of droplets in the buffer at 24 h (C) and 72 h (D) post

hydrogel formation. The data shows the size of the droplets that are present in the supernatant, all the droplets are below the size of one micron.

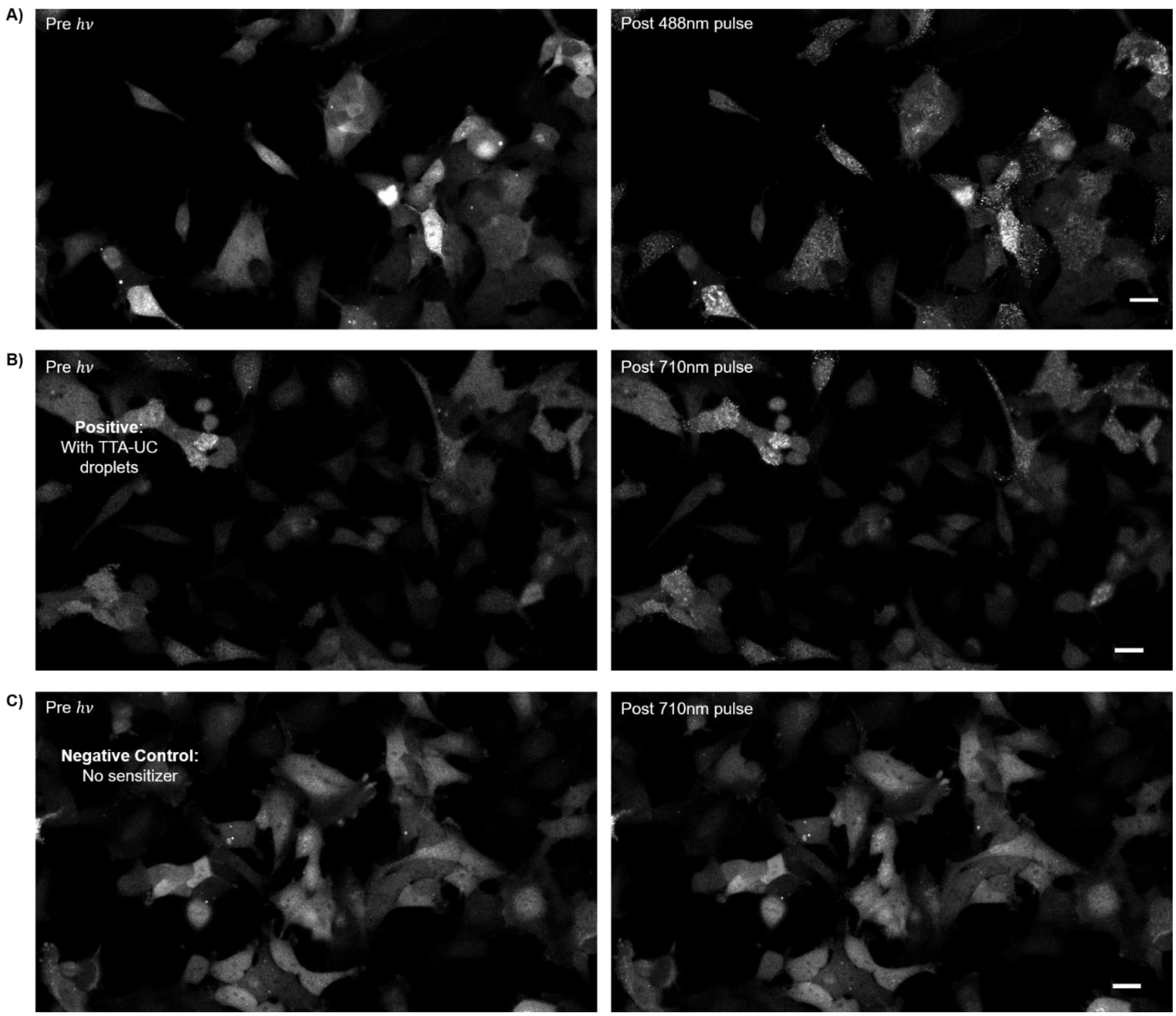

Figure S7. Upconversion-mediated optogenetic response in 2D. A) HeLa cells were directly irradiated using a pulse of blue light (λ_{ex} = 488 nm, 1 sec pulse), demonstrating that Cry2olig-mChy undergoes clustering in response to the high-energy stimulation. **B)** The cell media containing TTA-UC emulsion droplets (TIPS-An:PtTPTNP) was irradiated using a pulse of low-energy light (λ_{ex} = 710 nm, 1 sec pulse), demonstrating cell clustering in response to upconversion of the NIR light. **C)** The cell media containing control emulsion droplets (TIPS-An only) was irradiated using a pulse of low-energy light (λ_{ex} = 710 nm, 1 sec pulse), demonstrating lack of cell clustering in response to NIR light without upconversion. All images were obtained using the mCherry channel (λ_{ex} = 561 nm, and detection 570-640 nm) in a confocal microscope, clustering images were captured 10 min post irradiation. Scale bar, 25 µm.

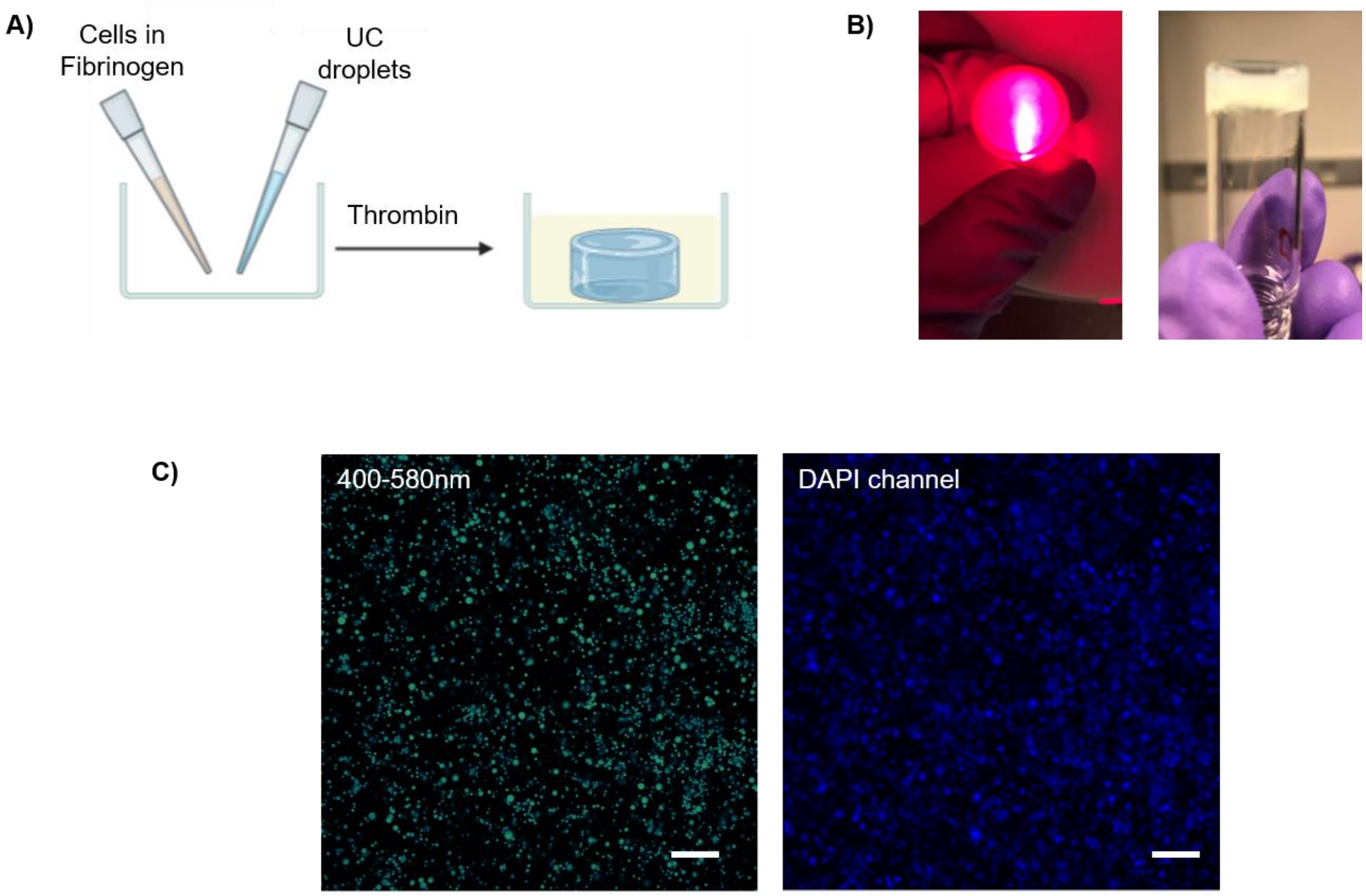

Figure S8. Design of upconversion biomaterial and cell encapsulation. A) Synthetic method to produce the hydrogel biomaterial doped with upconversion droplets. Cells resuspended in fibrinogen are mixed with the emulsion droplets and added to a well containing thrombin, which facilitates the crosslinking of fibrinogen to form the hydrogel. **B)** Red light (λ_{ex} = 637 nm) penetration through the free-standing gel. **C)** Upconversion luminescence emission within the biomaterials upon excitation with red laser (λ_{ex} = 637 nm) detected using the spectral detector (400-580 nm) of a confocal microscope shows the blue emission within the upconversion biomaterial. Additionally, hydrogels can be imaged with the DAPI channel (λ_{ex} = 405 nm and detection 425-475 nm). Scale bars, 25 µm.

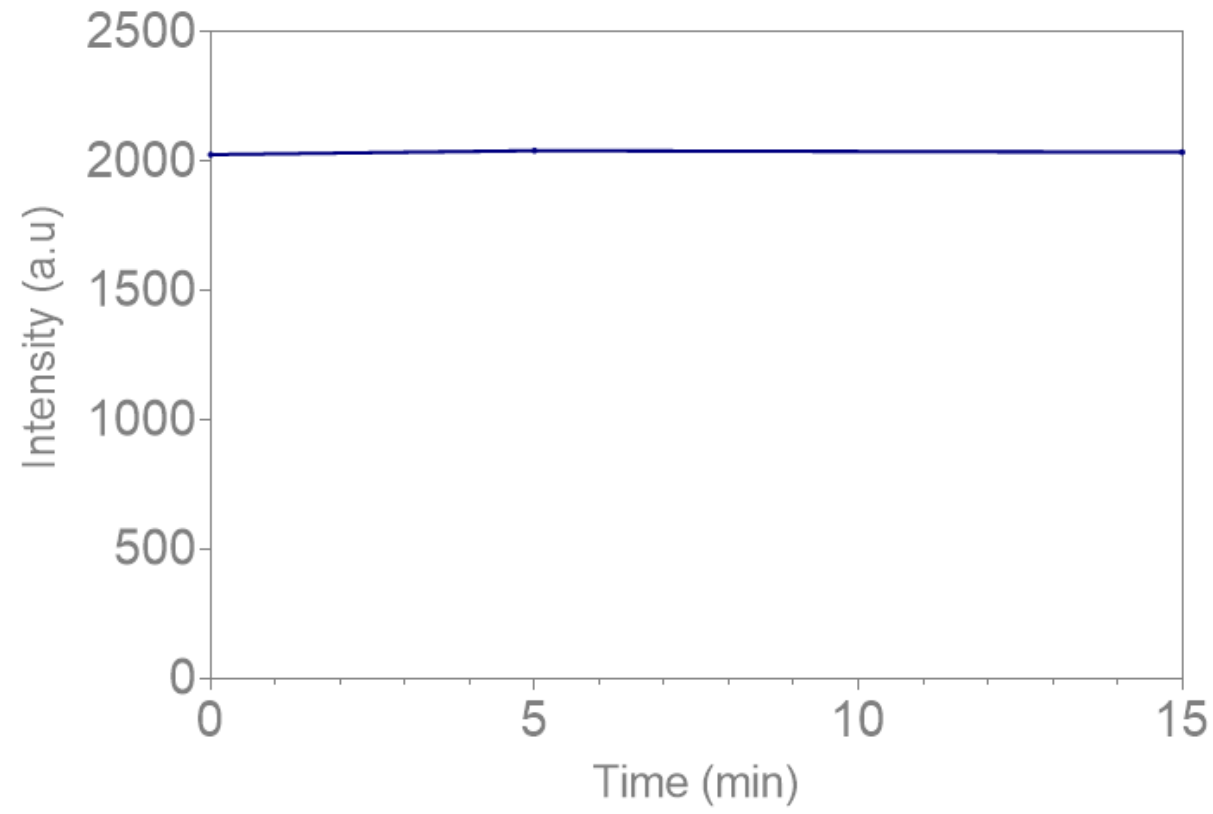

Figure S9. Stability of the upconversion system. Upconversion photoluminescence emission intensity over time, recorded at 450 nm, from the upconversion emulsions containing soybean oil, Pluronic F127, and the TIPS-An:PdTPTBP sensitizer and annihilator pair, embedded into the hydrogels. The sample was continuously irradiated with red laser diode (637 nm, 76 mW/mm^2) for 15 minutes without any change in photoluminescence.

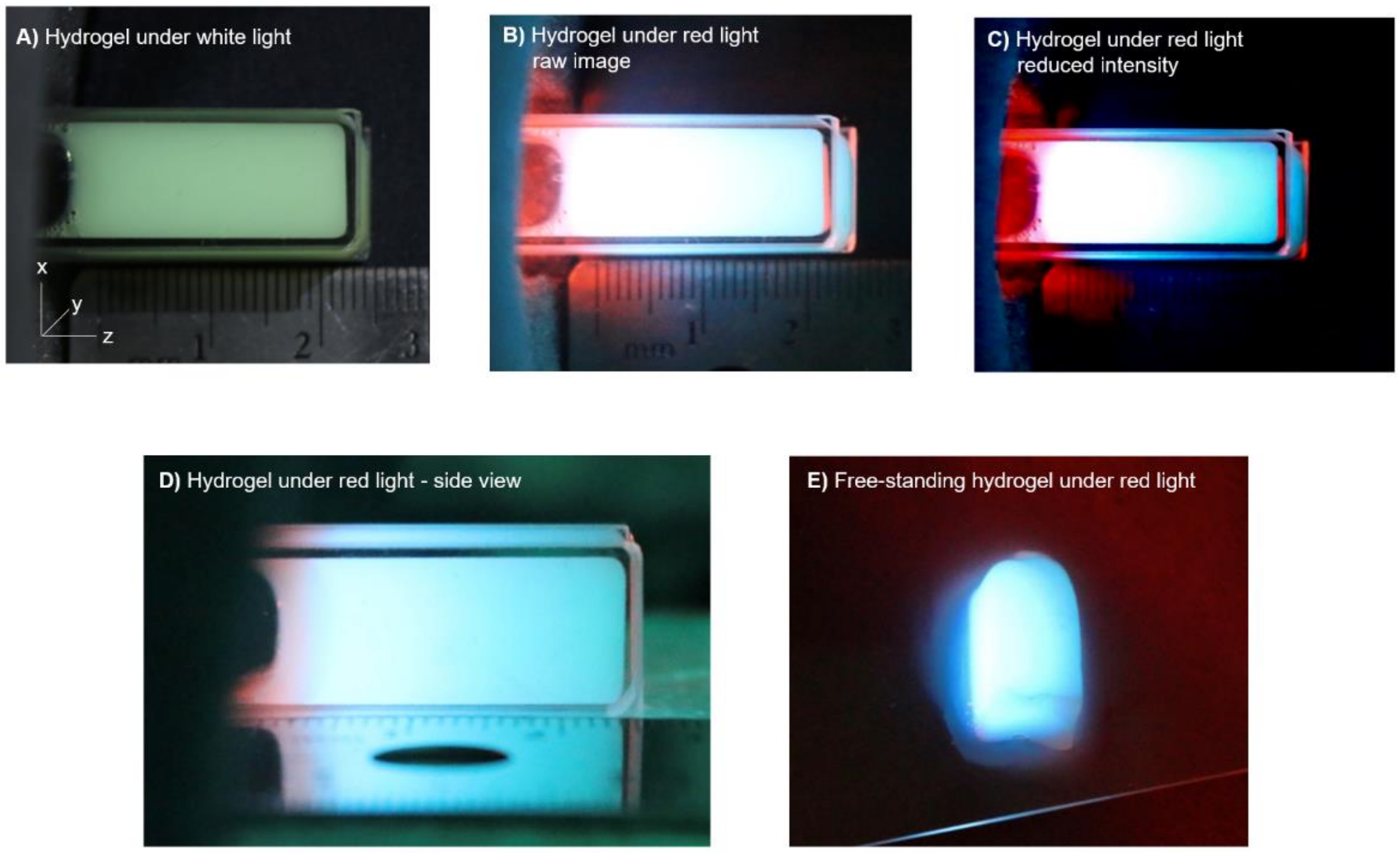

Figure S10: Qualitative evaluation of light penetration and emission from the opaque hydrogels. To demonstrate the ability of red light (640 nm) to penetrate the hydrogels, TTA-UC hydrogel with dimensions of (z = 2.5, x = 1, y = 1 cm) was made in a cuvette and photographed using a digital camera. **A)** Hydrogel under ambient light demonstrating a depth of 2.5 cm in the z axis. **B)** Red LED (640 nm) was placed one cm away from one side of the hydrogel, to irradiate the hydrogel through the z axis under ambient conditions. Upconverted blue light was visible, and photographs show scattered blue light emanating throughout the hydrogel. **C)** The brightness of image B was reduced to show the penetrating gradient in the gel as a function of distance from the light source. **D)** Side view of the same hydrogel demonstrating the penetration of the light and the brightness of the hydrogel. **E)** Free standing hydrogel upconversion upon red light excitation, demonstrated by taking the hydrogel out of the cuvette and placing it on a glass slide. Photographs were taken by using a cutoff filter in front of the lens of the digital camera.

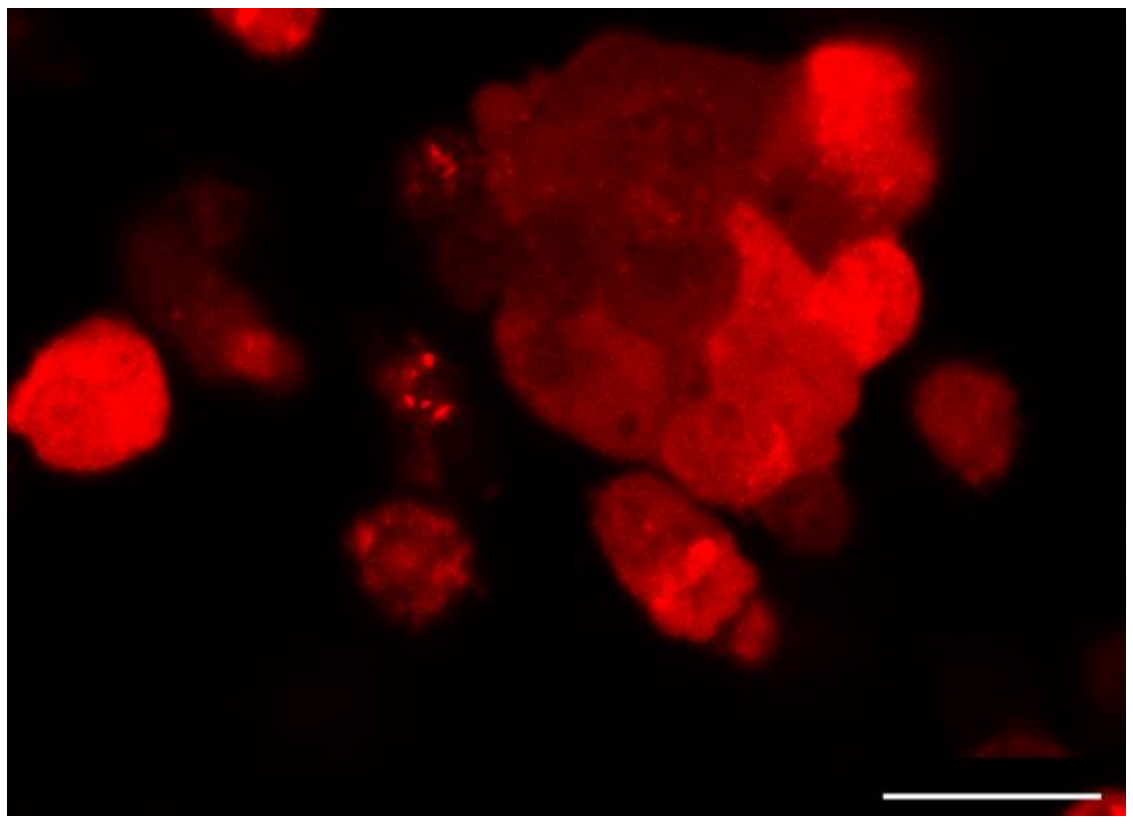

Figure S11. Durability of biomaterials. Representative confocal microscopy image of a cluster of live cells, 96 hours post encapsulation in the upconversion biomaterials using TIPS-An:PtTPTNP emulsion droplets. Scale bar, 25 µm.

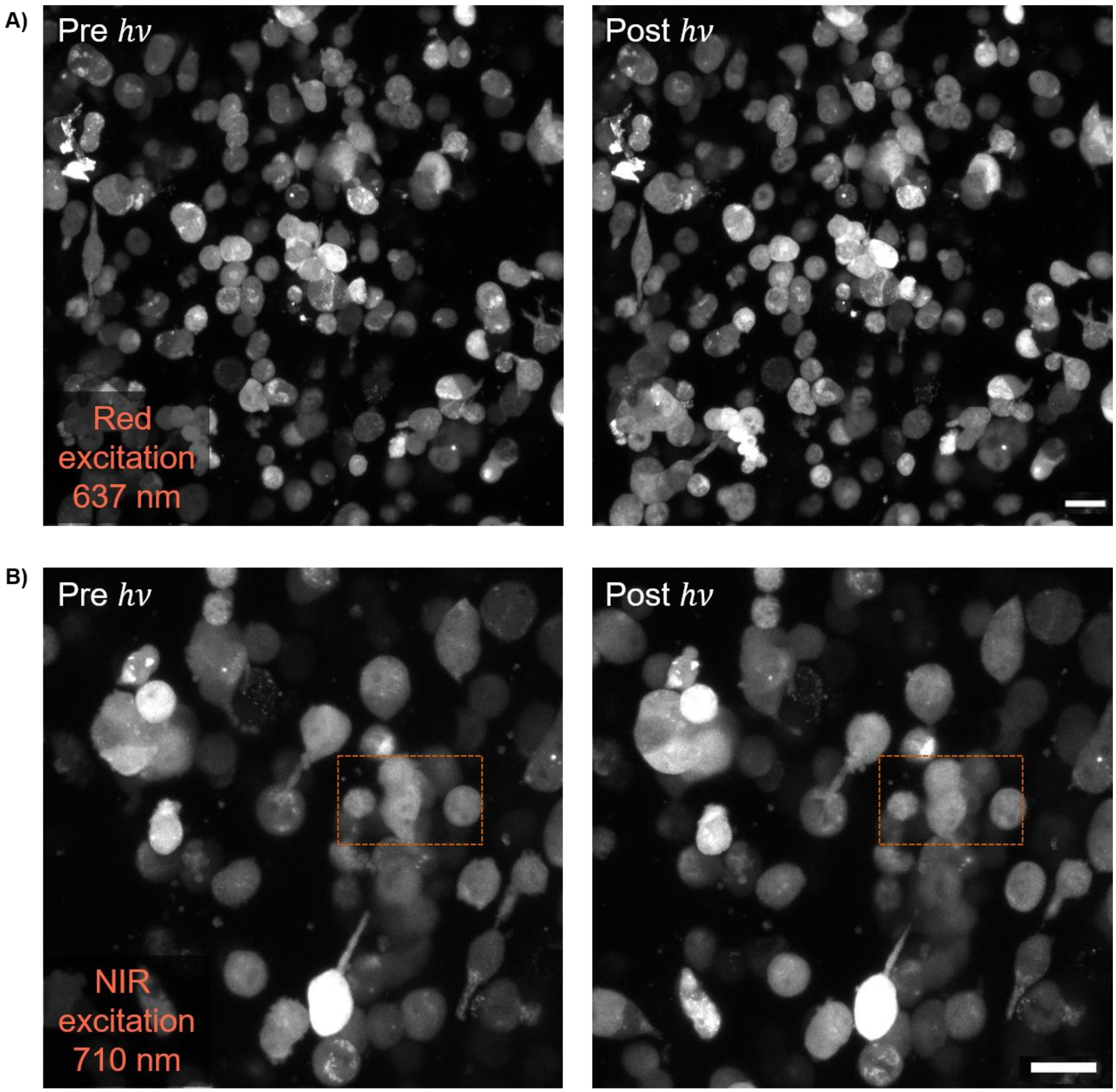

Figure S12. Red/NIR light does not trigger cells in 3D without the upconversion process. Representative max intensity projection of a confocal image of cells encapsulated in hydrogel with the control emulsion droplets (TIPS-An only, no sensitizer) two days post encapsulation before irradiation (left) and 10 min after irradiation (right) with: **A)** 637 nm laser excitation, showing no clustering of Cry2olig-mChy photoreceptors and **B)** 710 nm scanning laser excitation, focused on a small cluster of cells (in red frame), also showing no clustering. Scale bars, 25 µm.

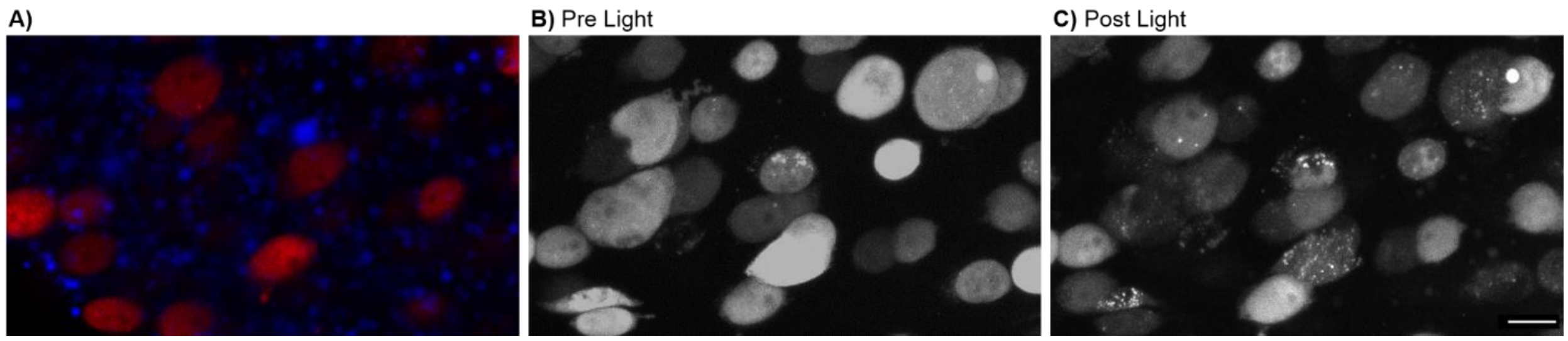

Figure S13. Stimulation of cells 24 hours post encapsulation. A) Microscopy image of cells (red) and emulsion droplets (TIPS-An:PtTPTNP, blue). **B)** mCherry signal before 710 nm laser stimulation. **C)** mCherry signal 10 min after 710 nm laser stimulation, demonstrating clear Cry2olig-mChy clustering. The images show maximum intensity projection. Scale bar, 25 µm.

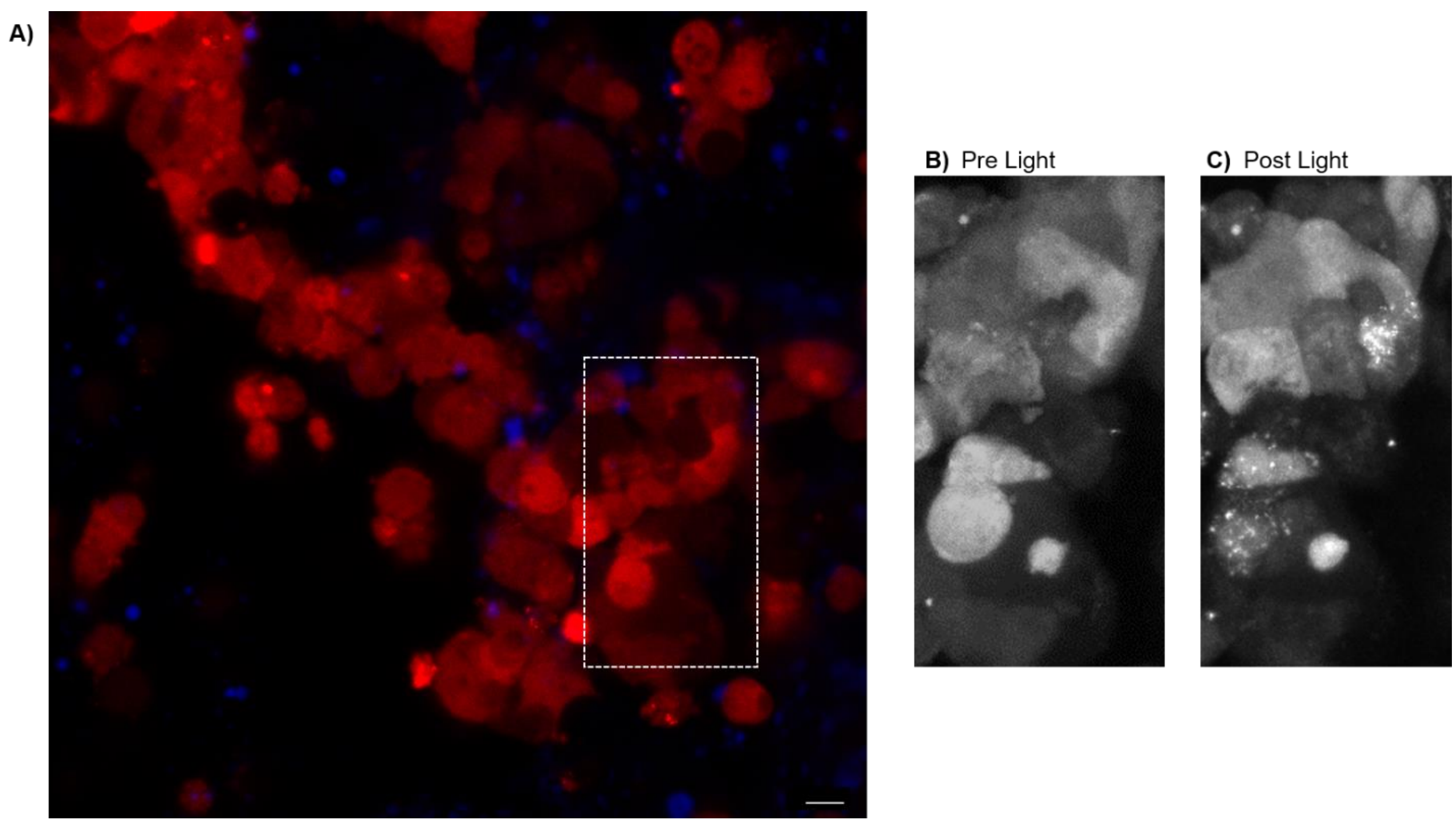

Figure S14. Stimulation of cells 72 hours post encapsulation. A) Microscopy imaging of cells (red) and emulsion droplets (TIPS-An:PtTPTNP, blue). **B)** mCherry signal before 710 nm laser stimulation. **C)** mCherry signal 10 min after 710 nm laser stimulation, demonstrating clear Cry2olig-mChy clustering. The images show maximum intensity projection. Scale bar, 25 µm.

METHODS

Materials

Unless otherwise stated, all starting materials were obtained from commercial sources and used without further purification. PdTPTBP was purchased from Santa Cruz Biotechnology and was used without further purification. PtTPTNP[2] and TTBP[3] were synthesized according to reported procedures. TIPS-An was synthesized as described below.

Synthesis of TIPS-Anthracene

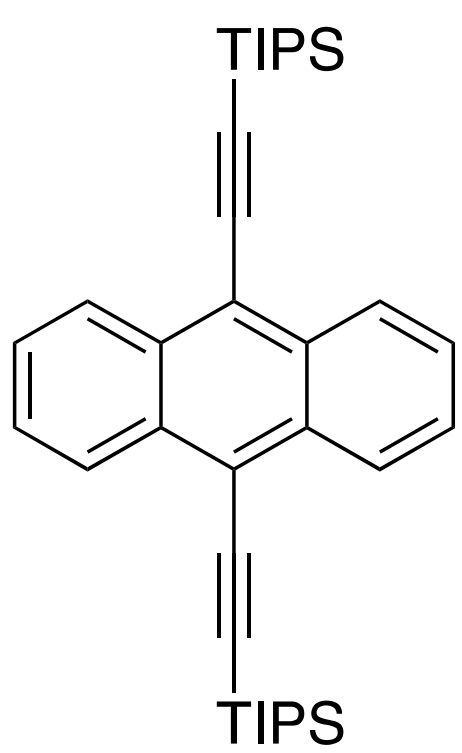

To a solution of TIPS-acetylene (2.7 g, 15 mmol) in anhydrous THF (15 mL) under argon at 0 °C was added n-butyl lithium (6 mL, 2.5M in hexane) over 5 min. The reaction was allowed to come to RT. After 1 h, anthraquinone (1 g, 5 mmol) was added in one portion and the reaction stirred overnight. The reaction was quenched and diluted with wet THF and poured into a rapidly stirred solution of stannous chloride dihydrate (33 g, 0.15 mol) in 10% hydrochloric acid (75 mL). After 1 h, the reaction was extracted with DCM (×3), the combined extracts washed with water (×3), brine and dried over $MgSO_4$. The solution was finally filtered through an inch of celite to remove residual stannous impurities. The residue obtained after concentration *in vacuo* was purified by column chromatography on silica gel eluting with hexane to give the title compound at a bright yellow solid (2.3 g, 84%). **^{1}H NMR** (500 MHz, $CDCl_3$) δ 8.67 (dd, *J* = 6.7, 3.2 Hz, 4H), 7.63 (dd, *J* = 6.7, 3.2 Hz, 4H), 1.34 – 1.25 (m, 42H).

Optical Properties

UV-visible absorption spectra were obtained on a Technologies Cary 60 UV−Vis Spectrophotometer. Photoluminescence spectra were obtained on a Horiba FluoroMax 4

Spectrofluorometer. Upconversion photoluminescence was measured in a Labsphere QE Integrating Sphere using an Ocean Optics QEPro Spectrometer.

Synthesis of Upconversion Emulsions

To a glass vial were added 100 µl of oil (soybean oil / D-limonene / TMB) and 900 µl buffer (20mM HEPES buffer in 0.9% saline) containing Pluronic F127 (10 wt%). The oil and buffer mixture was first gently agitated and then homogenized using a bead miller homogenizer (Bead Rupture 4, OMNI International). If the emulsion was not to be cultured with cells, then the resulting emulsion was used directly without further processing. If the emulsion was to be cultured with cells, then the resulting emulsion was filtered through a 0.22 µm PTFE syringe filter in sterile conditions to produce a sterile emulsion. For emulsions containing upconversion chromophores, a stock solution of TTA-UC oil was prepared: 0.1 mg of the sensitizer and 10 mg of the annihilator were dissolved in 100 µl chloroform followed by the addition of one ml of oil. The chloroform was naturally evaporated from the system by stirring the emulsions open to air for 8 h.

Synthesis of Upconversion Hydrogels

Hydrogels were made from Fibrinogen and Thrombin in 96-well plates. Fibrinogen was dissolved at 20 mg/ml in 20 mM HEPES buffer in 0.9% saline. Then 5 µl of the TTA-UC or control emulsions were mixed with 75 µl of the Fibrinogen solution. The resulting mixture was then added to a 96 well containing 20 µl of Thrombin (25 U/mL in 0.1% BSA in PBS) and gently mixed with a pipet three times to form the hydrogel.

Synthesis of Cell-Laden Upconversion Hydrogels

Hydrogels were formed in 96-well plates, for each well: CRY2olig-mChy stable HeLa cells were counted and 50,000 cells were resuspended in 35 µl Fibrinogen from human plasma (at 20 mg/ml; Sigma F3879), supplemented with 2.5 µl $CaCl_2$ at 50 mg/ml (2.5 mg/ml final concentration) and the indicated volume of emulsion. If needed, volume was adjusted to 40 µl with HEPES buffer (Gibco). The 40 µl, containing cells, fibrinogen and emulsion droplets was then added to a 96 well, containing 10 µl of Thrombin from human plasma (equals 0.25 units; Sigma T6884) and gently mixed with a pipet three times to form the hydrogel. Hydrogels were incubated for 30 min at 37 °C before adding 100 µl growth media supplemented with protease

inhibitor cocktail (1:200; Sigma P1860) to avoid digestion of the hydrogel by extracellular proteases.

Size Characterization of Upconversion Droplets

Droplets were imaged using a Nikon A1RMP laser scanning system based on an Eclipse TiE stand equipped with a 40x/1.3 PlanFluor oil-immersion objective. Samples were excited at 405 nm and emission was recorded from 425-475 nm. The scan zoom was set to give a pixel size of 0.316 µm. Z series were collected over a depth of 11 µm with a step size of 1 µm.

For analysis, maximum projections were generated and locally thresholded for particle analysis using Fiji. The Bernsen algorithm was used with a radius of 5 and a local contrast threshold of 30, and a binary watershed was performed to separate adjacent droplets. Diameter was determined as the maximum Feret diameter of each droplet.

Cell Culture

HeLa cells (obtained from American Type Culture Collection) were maintained in Dulbecco's Modified Eagle's Medium (Gibco) supplemented with 10% fetal bovine serum (Invitrogen), 1% GlutaMAX™ (Gibco), and 1% Penicillin-Streptomycin (Gibco), at 37 °C in a humidified 5% CO_2 atmosphere.

Stable HeLa Cell Line

To generate the stable HeLa cells, constructs containing the CRY2olig-mChy within the piggyBac transposon were introduced into cells by transfection and integrated using the piggyBac transposase.[4]

Cell Viability Assay

For viability assays testing biocompatibility of the emulsions, cells were plated in a 96-well white opaque plate format at 40,000 cells per well, allowed to adhere overnight, and then treated with emulsions at indicated concentrations for 1 h. These viability assays utilized the Cell-Titer-Glo 2.0 reagent (Promega, G9243) according to the manufacturer's instructions. Briefly, 100 µL of Cell-Titer-Glo 2.0 reagent, diluted at 1:1 with culture media, was added to each well containing 100 µl emulsion-containing media. Plates were gently agitated for 2 min and incubated at room temperature for 10 min in the dark. Luminescence was subsequently measured

using a Victor 5 plate reader (Perkin Elmer). The intensity of luminescence was normalized to that of cells with no emulsion particles added.

Cell viability in 3D hydrogels was measured in the same manner.

Fluorescence Microscopy Imaging and Irradiation Conditions

Images were taken on an Axio Observer 7 microscope (Zeiss) using 20X objective (0.8 N.A) with 2-by-2 pixel binning. For live-cell imaging, cells were imaged for a total of 70 min in 1 min intervals in a humidified, 37 ºC chamber with 5% CO_2. Samples were stimulated with red LED (637 nm) for 1 sec ranging from 11 to 55 mW (correlating to 24-126 mW/mm^2). Samples containing the upconversion emulsion clearly exhibit oligomerization at all light intensities, while samples containing the control emulsion did not exhibit oligomerization at all light intensities.. Total light exposure time for mCherry images was kept at 200 ms for each time point. Groups are n=12 imaging sites per condition, each imaging site includes ~100 cells. Images were processed using a Matlab script. Briefly, a mask of puncta was generated by top hat-filtering raw images with a circular kernel of radius 4 µm and thresholding absolute intensity. The puncta parameter represents the number of puncta in the field of view. Plot of time-lapse results and quantification of the cluster ratio in cells, was calculated by dividing the total cluster intensity by the total fluorescence of the whole cell.

Confocal Microscopy Imaging in 3D

Hydrogels were imaged and irradiated on a Nikon A1RMP laser scanning system based on an Eclipse TiE stand equipped with a 40x/1.3 PlanFluor oil-immersion objective. Upconversion photoluminescence was recorded using a spectral detector across 32 channels (402-588 nm). Hydrogels were taken out of the 96 wells at least one day after formation and placed on 25-mm round coverslips. For hydrogels including live-cell imaging, hydrogels on coverslips were placed in a humidified, 37 ºC chamber in 5% CO_2. For optogenetic activation a red laser diode was used (637 nm, 7.6 mW), or a scanning laser of NIR light (710 nm, 63 mW). Excitation time was 5 sec. For stimulation experiments, hydrogels were imaged before and after irradiation with mCherry channel.

References

[1] K. J. Fallon, E. M. Churchill, S. N. Sanders, J. Shee, J. L. Weber, R. Meir, D. R. Reichman, M. Y. Sfeir, D. N. Congreve, L. M. Campos, *J. Am. Chem. Soc.* **2020**.

[2] J. R. Sommer, A. H. Shelton, A. Parthasarathy, I. Ghiviriga, J. R. Reynolds, K. S. Schanze, *Chem. Mater.* **2011**, *23,* 5296.

[3] S. H. C. Askes, W. Pomp, S. L. Hopkins, A. Kros, S. Wu, T. Schmidt, S. Bonnet, *Small* **2016**, *12*, 5579.

[4] K. Yusa, R. Rad, J. Takeda, A. Bradley, *Nat. Methods* **2009**, *6*, 363.
